# Acute blood loss anemia aggravates endothelial dysfunction after acute myocardial infarction

**DOI:** 10.3389/fcvm.2025.1635293

**Published:** 2025-10-13

**Authors:** Isabella Solga, Aslihan Şahin, Vithya Yogathasan, Lina Hofer, Feyza Gül Celik, Amira El Rai, Mohammed Rabiul Hosen, Patricia Wischmann, Stefanie Becher, Amin Polzin, Norbert Gerdes, Christian Jung, Malte Kelm, Ramesh Chennupati

**Affiliations:** ^1^ Department of Cardiology, Pulmonology and Vascular Medicine, Medical Faculty and University Hospital, Heinrich-Heine University, Düsseldorf, Germany; ^2^ Molecular Cardiology, Heart Center Bonn, Department of Internal Medicine II, University Hospital Bonn, Bonn, Germany; ^3^ Cardiovascular Research Institute Düsseldorf (CARID), Medical Faculty, Heinrich-Heine-University, Düsseldorf, Germany

**Keywords:** anemia, endothelial dysfunction, nitric oxide, reactive oxygen species, red blood cells, myocardial infarction

## Abstract

**Background:**

Anemia is frequently observed in patients with acute myocardial infarction (AMI) and is known to be associated with poor prognosis. We recently demonstrated that acute blood loss anemia is associated with a compensatory increase in endothelial nitric oxide (NO)-dependent flow-mediated dilation (FMD) responses. However, the effects of acute anemia on systemic endothelial function after AMI remain unclear. In this study, we evaluated systemic endothelial function following AMI in an established murine model of acute blood loss anemia. We hypothesize that acute anemia aggravates systemic endothelial dysfunction (ED) after AMI.

**Methods and results:**

Acute anemia was induced in male C57BL/6J mice by repeated blood withdrawal for three consecutive days. Separate groups of anemic and non-anemic mice underwent AMI via left anterior descending artery (LAD) ligation (45 min), followed by reperfusion. Endothelial function was assessed using both *in vivo* and *in vitro* methods 24 h post-AMI. Impaired FMD (*in vivo*) and endothelium-dependent relaxation (EDR) responses were observed in the aorta, femoral, and saphenous arteries of AA mice compared to their respective control groups 24 h post-AMI. Analysis of oxidative products of NO in plasma revealed reduced nitrite and nitrate levels in acute anemia compared to controls 24 h post-AMI. Immunohistochemistry of aortic tissues from both anemic groups showed increased reactive oxygen species (ROS) product 4-Hydroxynonenal (4-HNE). Co-incubation of RBCs from anemic mice or anemic acute coronary syndrome (ACS) patients with aortic rings from wild-type mice demonstrated attenuated EDR responses. Supplementation with the ROS scavenger N-acetyl cysteine (NAC) for four weeks improved both *in vivo* and *ex vivo* EDR in acute anemic mice 24 h post-AMI.

**Conclusion:**

After AMI, acute anemia is associated with ROS-mediated severe endothelial dysfunction, which is partly mediated by RBCs. Antioxidant supplementation with NAC is a potential therapeutic option to reverse the severe ED in anemia following AMI.

## Introduction

Acute myocardial infarction (AMI) is one of the leading causes of sudden death worldwide due to a blockage in the coronary arteries resulting in necrosis of the myocardium (1–3). Anemia is a frequently diagnosed co-morbidity in patients with AMI, characterized by reduced hemoglobin levels, hematocrit, and a reduction in circulating red blood cells (RBCs). Many elderly patients with cardiovascular disease develop anemia during hospitalization due to blood loss from interventions or diagnostic procedures. This condition, leads to hospital-acquired anemia, adversely affects the prognosis during cardiovascular events (4). Anemia in association with acute coronary syndrome, stroke, or heart failure leads to poor cardiovascular disease (CVD) prognosis (5). Furthermore, anemic patients have a higher risk of major bleeding, arrhythmias, and heart failure after AMI, leading to morbidity and mortality (6, 7). However, the underlying mechanisms of how blood loss anemia influences vascular function are largely unknown.

The endothelium in arteries plays a fundamental role in regulating vascular tone by synthesizing and releasing an array of endothelium-derived relaxing factors, such as nitric oxide (NO) and endothelium-derived hyperpolarizing factor (EDHF). NO is generated from L-arginine by endothelial NO synthase (eNOS), which diffuses into vascular smooth muscle cells (VSMCs) and activates guanylate cyclase, resulting in cyclic guanosine monophosphate (cGMP)-mediated vasodilation (8). Reduced production of endothelium-dependent NO and increased levels of reactive oxygen species (ROS) and inflammation are associated with many forms of CVDs, including hypertension, coronary artery disease (CAD), and chronic heart failure (9–11). It is well known that increased reactive oxygen species (ROS) in the endothelium uncouple eNOS, leading to reduced nitric oxide (NO) production and, consequently, endothelial dysfunction (ED). ED is a hallmark of vascular dysfunction in acute myocardial infarction (AMI) and worsens the overall prognosis (12).

We recently showed that acute blood loss anemia is associated with severe red blood cell (RBC) dysfunction and a reduced circulating NO pools, accompanied by a compensatory enhancement in heart function and shear stress–mediated flow-mediated dilation (FMD) responses (13). These responses are compromised in chronic anemia (CA) due to increased oxidative stress (14). In the same study, we demonstrated that CA is associated with progressive ED in large arteries due to increased oxidative stress in the endothelium (14). However, the effect of blood loss anemia on endothelial function after AMI remains largely unknown, despite its potential translational relevance.

Studies have shown that red blood cells (RBCs) mediate ED in various disease states such as diabetes, hypocholesterolemia, preeclampsia, and anemia (15–17). We also recently demonstrated that RBCs in anemia, lose their cardioprotective properties in patients with ST-elevation myocardial infarction (STEMI) (18), further demonstrating the potential role of RBCs in cardiometabolic diseases. It is not clear how acute blood loss anemia affects vascular function after myocardial infarction, and the potential role of RBCs in this has never been investigated.

Therefore, in this study, we used a well-established acute blood-loss mouse model to study the effect of anemia on endothelial function after Ischemia-Reperfusion (IR) injury. Additionally, we investigated whether RBCs from anemic mice with AMI and anemic ACS patients affect endothelial function using co-incubation with aortic rings followed by endothelial function analysis. To our knowledge, this is the first study to evaluate systemic ED after AMI in acute blood-loss anemia murine model.

## Materials and methods

### Animals

All animal procedures used in the study were approved and performed in accordance with the ARRIVE (Animal Research: Reporting of *In Vivo* Experiments) II guidelines and authorized by LANUV (North Rhine-Westphalia State Agency for Nature, Environment and Consumer Protection) in compliance with the European Convention for the Protection of Vertebrate Animals used for Experimental and other Scientific Purposes. The approval numbers for the animal experiments are 84-02.04.2020.A073 and 84-02.04.2018.A234. C57Bl/6J (wildtype, WT) mice were obtained from Janvier Labs (Saint-Berthevin Cedex, France). Mice were housed in standard cages (constant room temperature and humidity, with 12 h light/dark cycles) and had free access to standard pelleted food and tap water.

### Anemia induction

For the experimental approach, we used four different groups of mice. The mice were divided into AA, and their respective non-anemic groups. AA was induced in 10- to 12-week-old male mice by repetitive mild blood withdrawal on three consecutive days, resulting in <20 g/L changes in hemoglobin (Hb). The blood was withdrawn from the facial vein under isoflurane anesthesia (3%). The amount of daily blood loss per mouse was adjusted to <15% of the total blood volume and was replaced by saline administration. The non-anemic group was age-matched to the anemic groups and underwent the same handling as the anemic mice (including puncture of the facial vein) without blood withdrawal.

### Induction of AMI

In a separate set of anemic and non-anemic groups of mice, acute myocardial infarction (AMI) was induced as described before (19), with some modifications. Briefly, mice were anesthetized with isoflurane, intubated, and ventilated with a tidal volume of 0.2–0.25 ml and a respiratory rate of 140 breaths per minute, using isoflurane (3%) and 30% O_2_ with a rodent ventilator. Body temperature was controlled and maintained at 37 °C throughout the surgical procedure. A left lateral thoracotomy was performed between the third and fourth rib, and the pericardium was exposed and dissected. Ischemia was induced by gently tightening a 7–0 surgical suture placed under the left anterior descending (LAD) artery. To ensure that the procedure was carried out correctly, in addition to the visible blanching of the apex, changes in the electrocardiogram (ECG; ST-segment elevation) were monitored. After 45 min, the ligation was removed, and the myocardium was reperfused for 24 h. Animals received buprenorphine (0.5 mg/kg BW) subcutaneously every 8 h until euthanasia.

### Supplementation of NAC

To investigate the potential role of reactive oxygen species (ROS) in mediating endothelial dysfunction, additional groups of mice were supplemented with 1% N-acetylcysteine (NAC; Sigma) administered through drinking water for 4 weeks. After 4 weeks, acute anemia was induced, and ischemia-reperfusion (IR) surgery was performed to induce AMI.

### Collection of blood from ACS patients

Blood was collected from Acute Coronary Syndrome (ACS) patients, including both ST-elevation myocardial infarction (STEMI) and non-ST-elevation myocardial infarction (NSTEMI) patients, with and without anemia, within 24 h post-infarction in EDTA tubes. The patients were diagnosed based on changes in the ECG. According to the World Health Organization (WHO) guidelines, adult male patients with hemoglobin (Hb) levels below 13.0 g/dl and adult female patients with Hb levels below 12.0 g/dl are considered anemic (20). All patients included in this study provided written consent and were recruited from the Department of Cardiology, Pulmonology, and Angiology at Düsseldorf University. Permission numbers for blood sample collections are 5481R, 2018-14, and 2018-47, as approved by the ethics committee of Düsseldorf University Hospital.

## *In vitro* studies with isolated aortic rings

### Solutions and drugs

Krebs-Ringer bicarbonate-buffered salt solution (KRB) contained (in mmol/L): 118.5 NaCl, 4.7 KCl, 2.5 CaCl_2_, 1.2 MgSO_4_, 1.2 KH_2_PO_4_, 25.0 NaHCO_3_ and 5.5 glucose. The KRB solution was continuously aerated with 95% O_2_/5% CO_2_ and maintained at 37 °C. Indomethacin (INDO; Sigma Aldrich,) was dissolved in ethanol. Acetylcholine (ACh), phenylephrine (PHE), N***^ω^***-nitro-arginine methyl ester (L-NAME) and sodium nitroprusside (SNP; all Sigma Aldrich) were dissolved in KRB solution.

### Wire-myograpgh experiments

Mice were euthanized under deep isoflurane anesthesia (4.5%). The thoracic aorta was dissected free from perivascular adipose tissue and cut into 2 mm aortic rings. The segments were then mounted in a wire myograph system [Danish Myo Technology (DMT), Aarhus, Denmark] and stretched to a force of 9.8 mN. The segments were allowed to normalize for 45 min with a periodic buffer change three times as described before (21, 22). Briefly, after 15 min of initial force adjustment to 9.8 mN, arterial segments typically lose 5% of their tension, which is readjusted to 9.8 mN. This step was repeated two more times within the 45 min normalization period. Arterial segments that do not show a stable tone were excluded from experiments. Additionally, periodic buffer change allows the tissue to reach an optimal physiological state of resting tension before the experimental protocol. Saphenous and femoral arteries were dissected free from surrounding fat and connective tissue and mounted in a wire myograph (DMT). Arterial segments (2 mm) were stretched to the optimal diameter at which maximal contractile responses to 10 μM norepinephrine (NA) could be obtained (23).

#### Contributions of NO, cyclo­oxygenase products to endothelium-dependent relaxation

In the first step, a concentration-response curve (CRC) for PHE (0.001–10 μM) and ACh (1 nM or 10 nM–10 µM) was generated in presence of the cyclooxygenase inhibitor indomethacin (INDO, 10 µM). Next, to evaluate the contribution of NO to the relaxation response in the arteries the CRC was repeated in the presence of both INDO and L-NAME (100 µM) a NOS inhibitor.

#### Sensitivity of vascular smooth muscle to NO

Additionally, the SMC sensitivity to NO was evaluated by performing a CRC in presence of INDO and L-NAME (100 µM), with the NO donor SNP (1 nM or 10 nM–10 µM).

### Co-incubation experiments with murine and human RBCs

To further investigate the effects of dysfunctional RBCs on endothelial-dependent relaxation responses in anemia, we performed co-incubation studies with murine and human RBCs. Blood was collected in EDTA tubes from non-anemic and anemic mice 24 h post-AMI, and additionally from ACS patients with and without anemia. The RBCs were isolated by centrifugation at 800× g for 10 min at 4 °C, and 40% hematocrit (hct) was prepared using KRB buffer. Aortic rings (2 mm) isolated from WT mice were co-incubated with the isolated RBCs for 2 h (murine RBCs) or 6 h (human RBCs) at 37 °C in a humidified incubator with 95% O_2_/5% CO_2_. RBCs were freshly prepared and used within one hour to ensure their stability. Humidity was maintained at around 95% to prevent evaporation. After the incubation, the aortic segments were mounted in the wire myograph system, and vascular function was evaluated.

### Flow-mediated dilation assessment

The C57BL/6J mice of week-old mice with and without anemia were used for the assessment of flow-mediated dilation (FMD) responses. The measurements were performed 24 h post -AMI. FMD responses were determined by using a Vevo 3,100 high-resolution ultrasound scanner using a 30–70 MHz linear transducer (Visual Sonics Inc., Toronto, Canada) as described previously (24). During the whole procedure/measurement mice were kept under 2%–2.5% isoflurane anaesthesia and a body temperature of 37 °C was maintained. To visualise the femoral artery, the transducer was placed at the lower limb and a vascular occluder (8 mm diameter, Harvard Apparatus, Harvard, Boston, MA, USA) was used to perform the dilation measurement (24). Initially baseline images of the vessel were recorded. To measure changes in the vessel diameter, first an occlusion phase was performed by inflating the cuff to 250 mmHg. During the occlusion the pressure was kept constant for 5 min and an image was taken every 30 s (Druckkalibriergerät KAL 84, Halstrup Walcher, Kirchzarten, Germany). Afterwards the cuff was released (reperfusion phase) for 5 min and again every 30 s an image was taken to determinate the FMD. Changes in vessel diameter were quantified as percent of baseline (%) = [diameter (max)/diameter (baseline)] × 100.

### Analysis of plasma nitric oxide oxidative (NOx) products

For the analysis of nitric oxide (NO) metabolites, blood was collected as described previously (25) in EDTA coated tubes from anemic and non-anemic mice. The whole blood was mixed with *N*-ethylmaleimide NEM/EDTA buffer (10 mM/2 mM, PBS, pH7.4) in a ratio (1:10 v/v blood sample) and centrifuged immediately at 800 *g* for 10 min. Plasma was separated and snap frozen until further use.

For nitrite analysis, Chemiluminescence Detector (CLD, Eco Physics GmbH) was used as described previously (25). Briefly, the reaction glass chamber of CLD was filled with reduction solution consisting of 45 mmol/L potassium iodide (KI) and 10 mmol/L iodine (I_2_) in glacial acetic acid and the chamber is heated and maintained at 60℃ during the procedure. The reaction chamber was continuously aerated with helium gas and connected to the CLD through an acid trapper (1M NaOH). First, a standard curve for NO release (reductive cleavage) was generated by injecting known concentrations of NaNO_2_ solution (0 nM–1 µM) into the reaction chamber. Next, plasma samples were injected into the reaction chamber. The NO release was calculated based on NO release from nitrite standards. Data analysis was performed by eDAQ Powerchrome software (eDAQ, Warsaw, Poland).

Nitrate in plasma samples was determined by using ENO-30 (AMUZA INC, San Diego, USA) which is based on the separation of nitrate and nitrite by high-performance liquid chromatography (HPLC) followed by the Griess reaction (26). First, plasma samples were deproteinized by mixing with ice-cold methanol (1:1 v/v) and then centrifuged at 10,000 g for 10 min at 4 °C. The supernatant (10 µl) was injected into the analyzer. The nitrate content of sample was calculated based on known nitrite standards. Data analysis was performed using Clarity chromatography software 8.2.02.094 (DataApex, The Czech Republic).

### Immunohistochemistry

Thoracic aortas from the anemic mice 24 h post AMI and respective non-anemic mice 24 h post AMI were fixed in formaldehyde (4%) for 2 h and stored in sucrose (30%) overnight. Afterwards the tissues were embedded in Tissue-Tek®, O.C.T and frozen until further use. Tissue sections (5 µm) were incubated overnight at 4 °C with rat anti-mouse CD31 (1:200 in blocking solution [(0.1% Saponin, 0.5% BSA, 0.2% fish gelatin in 1 × PBS) BD Biosciences] and goat anti-rabbit 4-hydroxynonenal (4-HNE; 1:200 in blocking solution; Abcam) antibodies. Next, the sections were incubated with respective goat anti-rat Alexa Fluor 488 (1:1,000 in blocking solution; ThermoFisher) and goat anti-rabbit Alexa Fluor 555 (1:1,000 in blocking solution; ThermoFisher) secondary antibodies. Additionally, autofluorescnece was quenched using Vector® TrueVIEW® Autofluorescence Quenching Kit (BIOZOL Vectorlabs). Prior to covering, a DAPI staining (1 mg/ml) was performed for 5 min and then the sections were mounted with VectaMount® Aqueous Mounting Medium (ThermoFisher) and imaged with Leica DM6 M microscope (Leica Microsystems). Both CD31- and 4-HNE-positive staining were determined within the region of interest based on pixel-level fluorescence intensity thresholds using software-assisted analysis [FiJi (v1.53c)]. The endothelial cell area (CD31-positive area) was expressed as the 4-HNE-positive area as a percentage (%) of the total endothelial cell area.

### Assessment of plasma inflammatory markers

Blood was collected from all experimental groups in EDTA tubes. RBC and plasma samples were prepared and snap-frozen. The samples were stored at −80 °C until further use. Plasma samples were used to assess inflammatory parameters in the different mice groups by using ELISA kits for VCAM-1 and ICAM-1 measurements (R&D Systems). Serum cytokine concentrations were quantified using the LEGENDplex™ Mouse Inflammation Panel (BioLegend, Cat. 740446). Assays were performed according to the manufacturer's instructions, with all samples analyzed in duplicate. Data were processed using the LEGENDplex Data Analysis Software.

### Flow cytometry analysis of ROS

To analyse the ROS production in RBCs. Blood was freshly collected and stained using the following antibodies and dyes: TER119 (Prod. Nr: 130-130-364, Miltenyi Biotec), 2′,7′-dichlorodihydrofluorescein diacetate (H2DCFDA)-B525-FITC (Thermo Fisher) and analysed using flow cytometer. The data was extracted using FlowJo software v10.5.3 and analysed using Graphpad Prism 10.0.

### Statistical analysis

In *ex vivo* studies, all concentration–response curves (CRCs) for contractile stimuli were expressed as absolute values. Relaxation responses were expressed as a percentage reduction from the level of contraction. Individual CRCs were fitted using a non-linear sigmoidal regression curve (GraphPad Prism 10.0). Sensitivity (pEC_50_) and maximal effect (Emax) are presented as means ± SEM. Comparisons between two groups were performed using unpaired *t*-tests. Two-way ANOVA followed by Bonferroni *post hoc* test was used for comparisons among multiple groups. Bonferroni correction is a method was used to adjust *p*-values to control for the increased risk of a Type I error (false positive).

## Results

### Acute anemia is associated with altered endothelial function post-AMI

**Figure 1:**
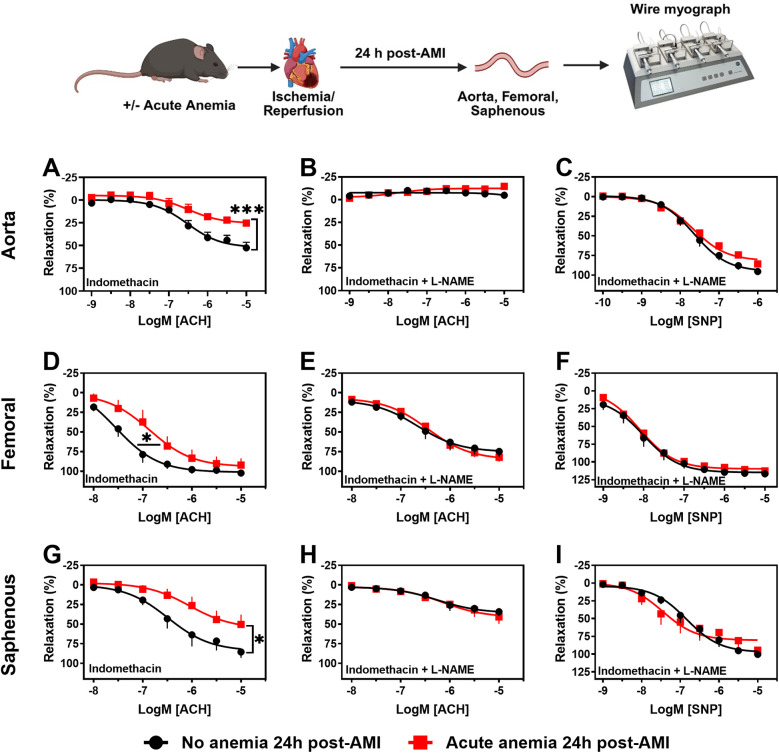
Acute blood loss anemia is associated with endothelial dysfunction in different vascular beds 24 h post-AMI. Arterial segments of aorta **(A–C)**, femoral **(D–F)** and saphenous arteries **(G–I)** were isolated from acute (red squares) and respective non-anemic (black circles) mice 24 h post-AMI. Arterial segments were pre-contracted using phenylephrine (PHE; 10 µM), and their relaxation responses to acetylcholine (ACH; 1 nM or 10 nM–10 µM) were assessed using wire myograph system. **(A,D,G)** Relaxation (%) in the presence of indomethacin (10 µM, a COX inhibitor). **(B,E,H)** Relaxation in the presence of indomethacin and L-NAME (100 µM, NOS inhibitor). Maximal endothelial-dependent relaxation response (*E*_max,_ %). **(C,F,I)** Relaxation responses to sodium nitroprusside (SNP, 1 nM or 10 nM–10 µM) in the presence of indomethacin and L-NAME. All values are mean values ± SEM (*n* = 8–10 per group). *, *p* ≤ 0.05; ***, *p* ≤ 0.001. Concentration-response curves (CRCs) were analysed by Two-Way ANOVA and Bonferroni's *post-hoc* test to compare acute anemic and non-anemic mice. Created in BioRender. Elster, C. (2025) https://BioRender.com/v2b27rs, licensed under Academic License.

To assess the effect of AA on vascular function 24 h post-AMI, contractile responses, and endothelium-dependent and -independent relaxation responses were measured in isolated aortic rings (large artery) using wire myograph. The contractile responses to phenylephrine did not significantly differ between AA mice and non-anemic mice 24 h post-AMI (Supplementary Table S1). However, the endothelium-dependent relaxation responses to acetylcholine in the presence of indomethacin were significantly reduced in AA mice (*E*_max_: 25.22 ± 2.88%; *p* = 0.0009) compared to the corresponding non-anemic group (*E*_max_: 52.45 ± 6.02%) of mice 24 h post-AMI (Figure 1A; Supplementary Figure S1A, Table S1). In the presence of the NOS inhibitor (L-NAME, 100 µM), relaxation responses were completely inhibited in both AA and non-anemic groups of mice (Figure 1B, Supplementary Table S1), proving that the relaxation responses are mediated by NO. SMC sensitivity to NO and endothelium-independent relaxation were assessed using an exogenous NO donor, sodium nitroprusside (SNP). All groups showed similar relaxation responses, indicating that SMC sensitivity to NO was similar in both groups (Figure 1C; Supplementary Table S1).

Endothelial function was also assessed in femoral (medium sized) and saphenous arteries (small sized) to investigate possible effects of anemia on different vascular beds. In both femoral and saphenous arteries, the contractile responses to phenylephrine did not significantly differ between AA mice and their respective control group 24 h post-AMI (Supplementary Tables S2, S3). In the presence of indomethacin, femoral arteries showed reduced sensitivity to acetylcholine-mediated relaxation responses in AA mice (pEC_50_: 6.86 ± 0.24; *p* = 0.04) compared to non-anemic mice (pEC_50_: 7.55 ± 0.20) 24 h post-AMI (Figure 1D; Supplementary Figure S1B, Table S2). In the small resistance saphenous artery, the endothelium-dependent relaxation responses to acetylcholine were significantly reduced in AA mice (*E*_max_: 50.36 ± 12.41%; *p* = 0.03) compared to the non-anemic group of mice (*E*_max_: 85.44 ± 7.59%) 24 h post-AMI (Figure 1G; Supplementary Figure S1C, Table S3). In both femoral and saphenous arteries, in the presence of L-NAME, the relaxation responses were similar between anemic and non-anemic groups (Figures 1E,H; Supplementary Tables S2, S3). Similarly, endothelium-independent relaxation responses to SNP did not differ between AA mice and their respective control groups' 24 h post-AMI (Figures 1F,I; Supplementary Tables S2, S3). These results hinting that AA is associated with reduced endothelium-dependent relaxation responses, which are mainly mediated by NO, despite the smooth muscle sensitivity to NO remains unchanged.

### Flow-mediated dilation responses are impaired in both acute anemic mice after AMI

**Figure 2:**
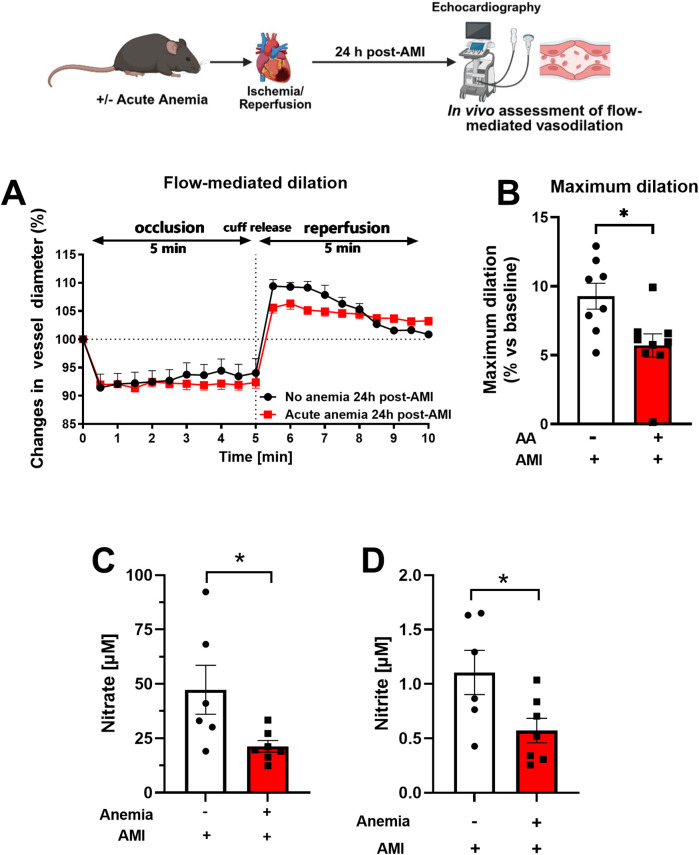
Acute blood loss anemia is associated with reduced flow-mediated dilation responses and reduced oxidative NO products 24 h post-AMI. **(A)** Changes in vessel diameter in acute anemic (red squares) and non-anemic group (black circles) of mice 24 h post-AMI. **(B)** Maximal FMD response (% ratio vs. baseline). **(C,D)** Plasma nitrate and nitrite levels in the in acute (red bar) and respective non-anemic group (white bar). The values are presented as means ± SEM. *, *p* ≤ 0.05. The average FMD in reperfusion phase was compared with the student t-test between the groups. Created in BioRender. Gerdes, N. (2025) https://BioRender.com/22v58jr, licensed under Academic License.

In addition to the detailed *ex vivo* assessment of vascular function in isolated arteries, we also assessed *in vivo* endothelial function by measuring FMD. AA mice showed significantly reduced FMD responses (5.70 ± 0.84%) compared to the respective non-anemic control group (9.27 ± 0.93%) 24 h post-AMI (Figures 2A,B). ED is often associated with reduced plasma oxidative nitric oxide (NOx) products. Therefore, we assessed the NOx products nitrite (NO_2_) and nitrate (NO_3_) in the plasma. AA mice showed significantly reduced plasma nitrate (Figure 2C) and nitrite (Figure 2D) levels compared to the respective non-anemic groups. These results clearly demonstrate that, in line with the reduced *ex vivo* endothelial-dependent relaxation responses and *in vivo* FMD responses, NO bioavailability is significantly reduced in AA mice compared to respective non-anemic mice. We previously showed that acute blood loss anemia is associated with enhanced FMD and cardiac output (13). The cardiac output becomes compromised after AMI (13). In this study, we observed a decreased cardiac output in the anemic mice 24 h post-AMI (Supplementary Figure S2G), whereas other cardiac functional parameters remained unaffected (Supplementary Figures S2B–F). These data conclude that acute blood loss anemia is associated with impaired endothelial function, which might contribute to decreased cardiac output.

### Acute anemia was associated with increased oxidative stress in the vessels

**Figure 3:**
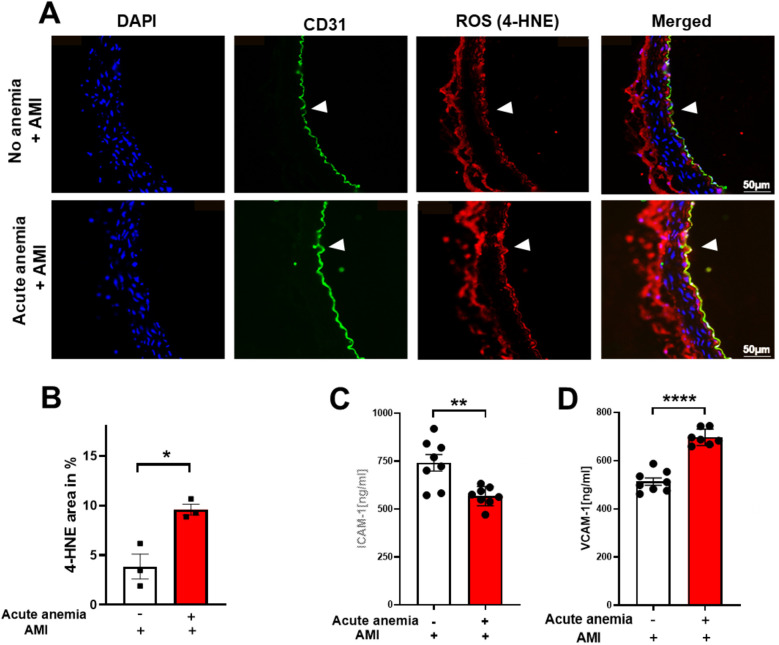
Acute blood loss anemia is associated with increased oxidative stress and inflammation 24 h post-AMI. Thoracic aortas isolated from acute anemic and non-anemic mice 24 h post-AMI. **(A)** Representative images and **(B)** quantification of sections stained with the endothelial cell marker CD31 (green) and the ROS marker 4-HNE (red). DAPI (blue) staining is used to detect nuclei. Arrowheads indicate the respective staining on the luminal side of the vessel. Results are representative of 3 (non-anemic) and 3 (acute anemic) independent experiments. Quantification is based on 4-HNE abundance (%) in the total endothelial cell area (CD31-positive area). **(C,D)** Plasma ICAM-1 and VCAM-1 levels in the acute (red bar) and non-anemic (white bar) groups of mice 24 h post-AMI. All values are presented as means ± SEM. *, *p* ≤ 0.05; **, *p* ≤ 0.01; ****, *p* ≤ 0.0001.

In our recent study, we showed that chronic anemia is associated with increased oxidative stress and endothelial activation (19). We further investigated the potential role of inflammation and reactive oxygen species (ROS) in the observed vascular dysfunction in AA 24 h post-AMI. To detect ROS in the vessels, we performed immunofluorescence staining for the ROS product 4-HNE. The aortic sections from AA mice 24 h post-AMI showed enhanced expression of 4-HNE levels compared to the non-anemic mice (Figures 3A,B). It is well known that ROS and inflammation concur with each other, so we also measured endothelial activation markers ICAM-1 and VCAM-1 in plasma. VCAM-1 was significantly increased, whereas ICAM-1 levels were decreased in AA mice compared to their respective non-anemic mice 24 h post-AMI (Figures 3C,D). Additionally, pro-inflammatory cytokine IL-1α was significantly increased in the plasma of AA mice compared to their respective non-anemic mice 24 h post-AMI (Supplementary Figure S3). These results suggest that AA was associated with increased oxidative stress and mild endothelial inflammation after AMI.

### ROS scavenger N-acetyl cysteine treatment reversed the endothelial dysfunction in acute anemic mice

**Figure 4:**
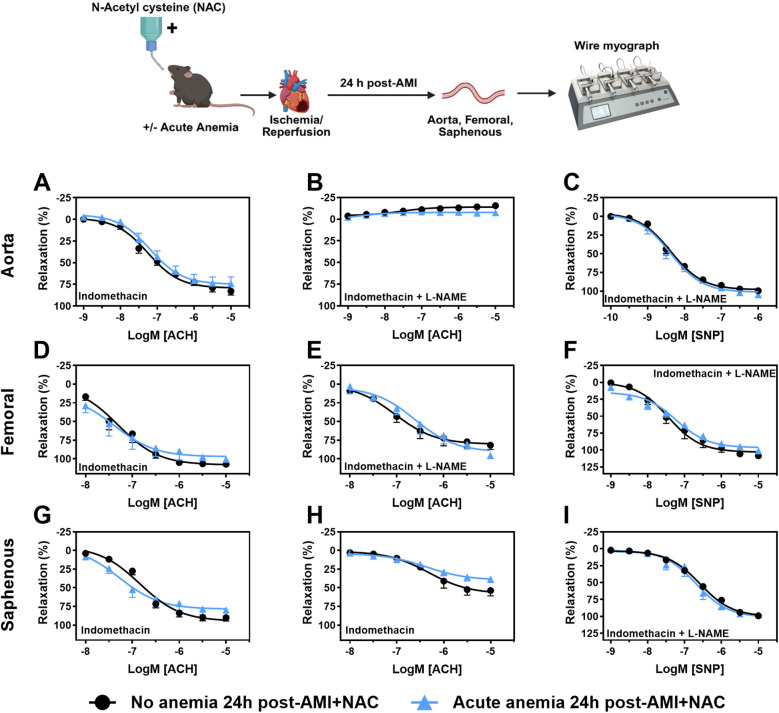
NAC supplementation improves endothelial-dependent relaxation response in acute blood loss anemia 24 h post-AMI. Arterial segments of aorta **(A–C)**, femoral **(D–F)** and saphenous arteries **(G–I)** were isolated from acute (light blue triangles) and non-anemic mice (black circles) 24 h post-AMI. Arterial segments were pre-contracted using phenylephrine (PHE; 10 µM), and their relaxation responses to acetylcholine (ACH; 1 nM or 10 nM–10 µM) were assessed using wire myograph system. **(A,D,G)** Relaxation (%) in the presence of indomethacin (10 µM, a COX inhibitor). **(B,E,H)** Relaxation in the presence of indomethacin and L-NAME (100 µM, NOS inhibitor). Maximal endothelial-dependent relaxation response (*E*_max,_ %). **(C,F,I)** Relaxation responses to sodium nitroprusside (SNP, 1 nM or 10 nM–10 µM) in the presence of indomethacin and L-NAME. All values are mean values ± SEM (*n* = 8–10 per group). Concentration-response curves (CRCs) were analysed by Two-Way ANOVA and Bonferroni 's *post-hoc* test to compare acute anemic mice and non-anemic mice. Created in BioRender. Gerdes, N. (2025) https://BioRender.com/wlrc3bx, licensed under Academic License.

From our data, it is evident that ROS mediates ED in AA mice. To further confirm this, we supplemented mice with NAC, 24 h after AMI, vascular function was assessed in isolated aorta, femoral and saphenous arteries. The contractile responses to phenylephrine were not altered between AA mice and the respective non-anemic group in the isolated aorta (Supplementary Table S1), femoral (Supplementary Table S2) and saphenous arteries (Supplementary Table S3). Next, we assessed endothelium-dependent and -independent relaxation responses in AA mice and the respective control group 24 h post-AMI. As expected, in AA mice, the endothelium-dependent relaxation responses were improved in all types of arteries with NAC treatment (Figures 4A–G; Supplementary Figures 4A–C, Tables S1–S3). The endothelium-independent relaxation to SNP was preserved in both anemic groups and all three types of arteries (Figures 4C–I; Supplementary Tables S1–S3). These results demonstrate that NAC treatment effectively reverses ED in acute anemic mice by improving both contractile and endothelium-dependent relaxation responses, indicating the critical role of ROS in mediating ED.

### ROS scavenger N-acetyl cysteine supplementation improved flow-mediated dilation responses in acute anemic mice after AMI

**Figure 5:**
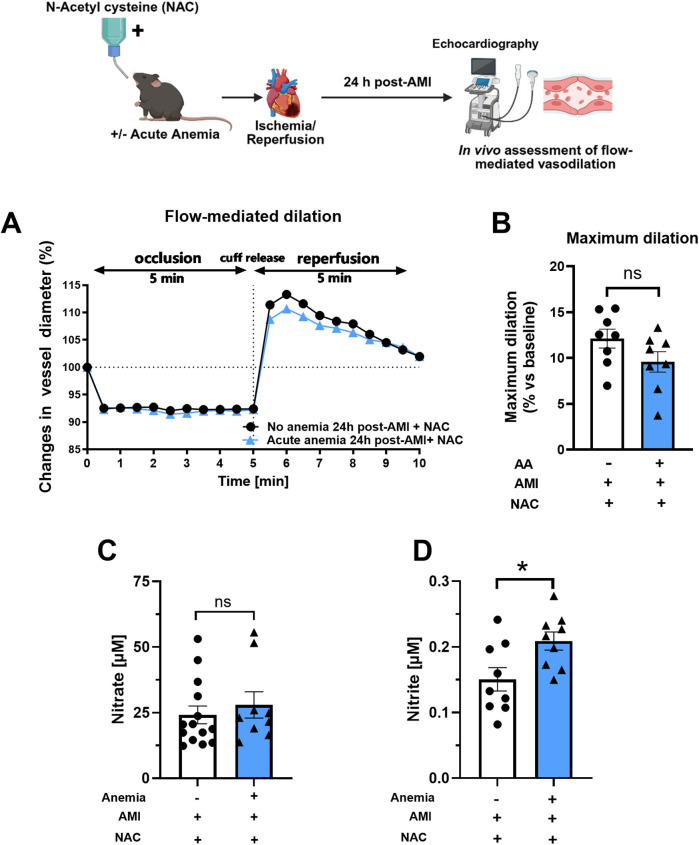
NAC treatment improves flow-mediated dilation (FMD) response in acute anemic mice 24 h post-AMI. **(A)** Changes in vessel diameter in acute (light blue triangles) and respective non-anemic groups (black, circles) of mice 24 h post-AMI. **(B)** Maximal FMD response (% ratio vs. baseline). **(C,D)** Plasma nitrate and nitrite levels in the in acute (light blue bar, triangles) and respective non-anemic group (white bar, circles). The values are presented as means ± SEM (*n* = 8–9 per group). *, *p* ≤ 0.05; ns, not significant. The average FMD in the reperfusion phase was compared with the student's *t*-test between the two groups. Created in BioRender. Gerdes, N. (2025) https://BioRender.com/tlpyhn8, licensed under Academic License.

We further assessed the FMD responses in AA mice and their respective non-anemic groups 24 h post-AMI. In line with *ex vivo* data, after NAC supplementation, both AA mice showed improved FMD responses (Figures 5A,B; Supplementary Figures S5A,B). This further confirms that ROS mediates vascular dysfunction in both anemic groups after AMI. Additionally, after NAC supplementation, both nitrite and nitrate levels improved in AA mice compared to the respective non-anemic groups 24 h post-AMI (Figures 5C,D). These results conclude that NAC supplementation improves ED and the bioavailability of NO.

### Anemic RBCs from mice and ACS patients promote endothelial dysfunction

**Figure 6:**
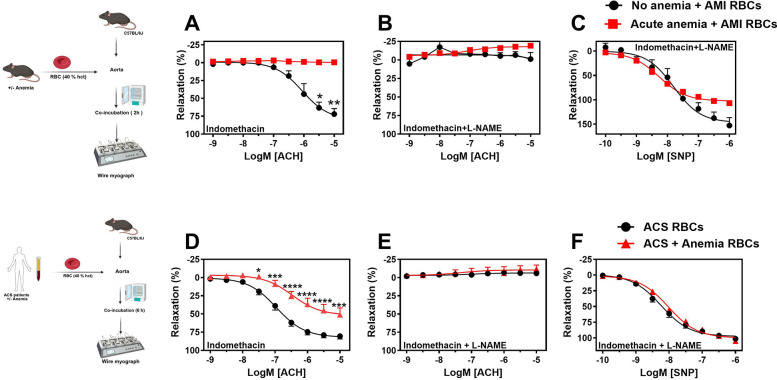
RBCs from anemic mice 24 h post-AMI and anemic ACS patients induce endothelial dysfunction. **(A–D)** RBCs were isolated from non-anemic (black circles) and anemic mice (red squares) 24 h post-AMI. **(E–H)** RBCs were isolated from ACS patients without anemia (black circles) and with anemia (red triangles). Haematocrit (40%) was prepared in a KREBS buffer. Aortic rings from WT mice were incubated with haematocrit either 2 (mice) or 6 (humans) hours and mounted in wire myograph system. **(B,F)** Relaxation responses (%) to acetylcholine (ACH; 1 nM or 10 nM–10 µM) in the presence of indomethacin (10 µM, COX inhibitor) in aortic rings incubated with mice RBCs. **(C,G)** Relaxation responses to acetylcholine in the presence of indomethacin (10 µM, COX inhibitor). **(D,H)** Relaxation responses to sodium nitroprusside (SNP, 10 nM–10 µM) in the presence of indomethacin and L-NAME. Values are shown as means ± SEM. *n* = 4 for mice (per group). ACS patients without anemia: *n* = 14, ACS patients with anemia: *n* = 11. *, *p* ≤ 0.05; **, *p* ≤ 0.01; ***, *p* ≤ 0.001: ****, *p* ≤ 0.0001. CRCs were analysed by Two-Way ANOVA and Bonferroni's *post-hoc* test. Created in BioRender. Elster, C. (2025) https://BioRender.com/inu8r2p, licensed under Academic License.

Previous studies have demonstrated that RBCs induce ED in various cardiometabolic diseases (20). Given this, we investigated the potential role of anemic RBCs in mediating ED after AMI. First, we incubated RBCs from anemic and non-anemic mice 24 h post-AMI, co-incubated (2 h at 37 °C) with WT aortic rings, and assessed the vascular responses. The endothelium-dependent relaxation responses to acetylcholine in the presence of indomethacin were completely inhibited in the aortic rings incubated with anemic RBCs compared to non-anemic mice (Figure 6A). The relaxation responses in the presence of L-NAME were completely inhibited in both groups (Figure 6B), indicating that relaxation responses are entirely mediated by NO in these vessels. In addition, the relaxation responses to SNP were mildly abrogated in anemic RBCs-incubated aortic rings compared to the non-anemic group (Figure 6C). These results suggest that RBCs from anemic mice 24 h post-AMI mediate endothelial dysfunction (ED).

We further investigated whether soluble factors released from RBCs or direct contact of RBCs promote ED in isolated aortic rings. Interestingly, the transwell separation experiment showed that anemic RBCs from mice 24 h post-AMI induce ED through direct contact rather than via the secretome (Supplementary Figures S6A–C). In addition, RBCs from anemic mice exhibited a modest increase in ROS production compared with non-anemic mice 24 h post-AMI (Supplementary Figure S7E). Taken together, these findings suggest that ROS released from RBCs of acute anemic mice 24 h post-AMI contribute to ED.

We also examined whether RBCs from anemic ACS patients induce ED. Aortic rings incubated with RBCs from anemic ACS patients displayed reduced endothelium-dependent relaxation responses compared with rings incubated with RBCs from ACS patients without anemia (Figure 6D). These relaxation responses were endothelium-dependent and mediated by NO (Figure 6E). Endothelium-independent relaxation responses to SNP were similar in both groups (Figure 6F). Furthermore, treatment of RBCs from anemic patients with NAC (1 µM) followed by co-incubation with aortic rings improved relaxation responses (Supplementary Figures S9A–C). These findings demonstrate that RBCs from anemic ACS patients induce ED by impairing NO-mediated relaxation responses, and that ROS produced by RBCs may contribute to this effect.

## Discussion

The current study investigated the effect of acute blood loss anemia on vascular function after AMI revealing several key findings: (1) AA is associated with reduced endothelial NO-dependent relaxation responses *ex vivo* and impaired *in vivo* flow-mediated dilation responses 24 h post-AMI, (2) Plasma nitrate and nitrite levels were significantly reduced in AA mice 24 h post-AMI, (3) AA was associated with increased production of ROS in the vessels, (5) NAC treatment improved endothelial function in AA mice 24 h post-AMI, (6) RBCs from anemic mice 24 h post-AMI and ACS patients with anemia induced endothelial dysfunction in murine aortic rings, highlighting the potential role of RBCs in ED.

Several comorbidities lead to adverse outcomes after AMI, particularly anemia, which is clinically presented in elderly patients during admission, hospitalization, or post-AMI (4, 27). The impact of blood loss anemia on vascular function is critical, as it often results from necessary medical procedures such as blood sampling and catheter insertion, which are unavoidable in the clinical setting (27). In this study, we utilized an acute blood loss anemic mouse model that parallels the clinical scenario of hospitalized patients. Our previous research indicated that while this model exhibited mild anemia without severe complications like edema, it also provided insights into the relationship between anemia and vascular health post-AMI (13, 14).

In our previous study we showed that AA (without AMI) is associated with compensatory increased FMD responses (13). In addition, previous clinical studies demonstrated that anemia without comorbidities results in increased FMD responses in healthy volunteers, whereas anemia in combination with chronic kidney disease (CKD) or diabetes worsens endothelial function and thus FMD responses (28–30). The key findings of this study are that anemia exacerbates endothelial dysfunction and impairs FMD responses after AMI due to increased oxidative stress and endothelial inflammation. This study is pioneering in elucidating how anemia, particularly in the presence of AMI, detrimentally affects endothelial function following AMI. In addition, isolated aorta, femoral, and saphenous arteries showed impaired acetylcholine-induced endothelium dependent relaxation responses. These responses were similarly reduced anemic mice compared to control mice in the presence of eNOS inhibitor (L-NAME), indicating that NO-dependent relaxations are affected in all sizes of vessels. Furthermore, smooth muscle sensitivity to NO remained similar between anemic and non-anemic mice, demonstrating that smooth muscle responsiveness to nitric oxide is not affected in anemia. Instead, the data suggest that nitric oxide release from the endothelium is impaired. The impaired NO-dependent relaxation responses are also reflected in abrogated *in vivo* FMD responses, which are mainly mediated by NO.

Recent studies have elucidated the critical role of endothelial nitric oxide synthase (eNOS) uncoupling in the development of ED across various pathologies (31). Under oxidative stress conditions, reactive oxygen species (e.g., O_2_^−^) can react with nitric oxide (NO) to form peroxynitrite (ONOO^−^), leading to eNOS uncoupling and subsequent lipid peroxidation, which contributes to vascular damage (31, 32). The uncoupling of eNOS not only disrupts the production of NO but also shifts the balance toward the generation of superoxide, further exacerbating oxidative stress and impairing endothelial function (33). In our investigation, we observed elevated levels of the reactive oxygen species product 4-HNE in the vasculature of anemic mice, suggesting a significant role of ROS in compromising endothelial integrity and NO availability. Moreover, the concomitant reduction in NO oxidative products, such as nitrite and nitrate, in these mice post-acute myocardial infarction, underscores the diminished availability of NO in the context of oxidative stress.

In this study, we demonstrated that RBCs from anemic mice 24 h post-AMI showed an increase in ROS. Although the increase was modest, the large number of circulating RBCs might release ROS and contribute to ED, particularly at the microcirculatory level. However, we cannot exclude other potential sources of reactive oxygen species (ROS) that may be upregulated in the vasculature as a result of anemia and AMI. For instance, mitochondrial dysfunction in endothelial cells is a plausible contributor (34). Our previous findings also indicated mild hemolysis in this mouse model (13), which releases free heme and iron, both of which can catalyze ROS formation via Fenton reactions (35). Additionally, we observed endothelial activation, which may stimulate NADPH oxidases and further enhance ROS production (36). While the exact source of ROS remains unclear, we speculate that multiple pathways are likely involved rather than a single predominant source.

Several studies have established that RBCs play a significant role in mediating ED in various pathological conditions, including diabetes mellitus and hypercholesterolemia, primarily due to elevated levels of arginase 1 and increased reactive oxygen species (ROS) formation in endothelial cells (16, 17). Our co-incubation experiments involving RBCs from anemic mice 24 h post-AMI and from anemic ACS patients with murine aortic rings revealed a notable induction of ED in these aortic rings. Interestingly, co-incubation of wild-type mouse aortic rings with anemic RBCs that were treated with N*ω*-Hydroxy-nor-L-arginine acetate, an arginase inhibitor, did not yield any significant improvement in endothelial function. These results suggest that a distinct mechanism mediates ED in anemia, highlighting the need for further research in this context.

## Study limitations

Our blood-loss anemia mouse model may not capture the multifactorial causes of anemia commonly observed in AMI patients (e.g., anemia of inflammation, nutritional deficiencies, or chronic disease). We acknowledge this limitation and emphasize the need for future studies using different anemic mouse models to determine whether our findings are specific to this type of anemia or applicable to a broader spectrum of anemic conditions. We used an established mouse model of blood loss anemia to study endothelial function 24 h after AMI in the context of acutely-acquired anemia (13). However, since the anemia was induced prior to AMI and continued for 24 h after AMI, it may not fully recapitulate all aspects of hospital-acquired anemia in AMI patients that by definition arises only after the onset of symptoms and admission to the hospital. Furthermore, our study was designed to assess the acute effects of anemia on endothelial function 24 h after AMI in order to capture the early changes in vascular responses. Short-term evaluation does not address the potential long-term consequences of anemia on vascular remodelling (e.g., progression of atherosclerosis) or its association with adverse clinical prognosis (e.g., reinfarction risk). We also emphasize the need for long-term follow-up studies to clarify whether acute anemia after AMI has a persistent impact on vascular health and cardiovascular outcomes. Such studies could provide important insights into the prognostic implications of anemia in AMI patients and inform tailored therapeutic strategies. In addition, our unpublished proteomic analysis revealed no change in total eNOS expression in the aorta of acute anemic mice compared with non-anemic controls 24 h after AMI, which may suggest possible eNOS uncoupling due to increased oxidative stress. However, assessment of the phosphorylated form of eNOS (e.g., ser1177, Thr495) would provide more precise information regarding its activity, and this requires further investigation. Additionally, short-term, acute-phase NAC administration should be tested in future studies to determine whether rapid antioxidant and anti-inflammatory modulation can improve endothelial function, reduce infarct size, and enhance outcomes in AMI patients. Our cytokine analysis revealed significantly increased plasma IL-1α levels in anemic mice compared with non-anemic mice 24 h post-AMI. The potential role of IL-1α in mediating ROS production, or conversely ROS-mediated IL-1α stimulation, warrants further investigation.

Despite these limitations, our study demonstrated that endothelial dysfunction (ED), mediated by anemia, becomes even more pronounced following acute myocardial infarction (AMI). These results also suggest that the deterioration of endothelial function may play a significant role in AMI and could contribute to worse outcomes in anemic patients. Our findings highlight anemia and anemia-related ED as critical therapeutic targets in this patient population. Furthermore, our study revealed that N-acetylcysteine (NAC) supplementation completely reversed ED, underscoring the important role of reactive oxygen species (ROS) formation in anemia. NAC is well known for its antioxidant properties and has been proposed as a potential treatment in clinical settings, particularly for conditions such as diabetic cardiomyopathy, coronary artery disease (CAD), heart failure, and AMI due to its cardioprotective effects (37). In our study, we demonstrated the potential benefits of NAC on endothelial function in the context of anemia. Based on these findings, we strongly believe that NAC supplementation may improve systemic vascular function in blood loss anemia. Given that NAC is safe and widely used, clinical trials should be considered to evaluate its therapeutic potential in this setting.

## Conclusions

Our research demonstrates a significant association between acute blood-loss anemia and decreased nitric oxide production following acute myocardial infarction, likely attributed to heightened inflammation and increased reactive oxygen species levels. Notably, we found that red blood cells derived from anemic ACS patients contribute to compromised endothelium-dependent relaxation, suggesting a critical role of these anemic RBCs in endothelial dysfunction. Additionally, our experiments revealed that administration of the ROS scavenger N-acetylcysteine in anemic mice effectively restored relaxation responses, implying the potential for ROS scavenging therapies to reinstate endothelial function in anemic conditions post-AMI.

## Data Availability

The raw data supporting the conclusions of this article will be made available by the authors, without undue reservation.
