# Supplementary material for: Acute blood loss anemia aggravates endothelial dysfunction after acute myocardial infarction
Source: Front Cardiovasc Med. 2025 Oct 13;12:1635293. doi: 10.3389/fcvm.2025.1635293 (PMC12554764; doi:10.3389/fcvm.2025.1635293)
Supplement: Supplementary file 1 [file Presentation1.pptx]

## Slide 1
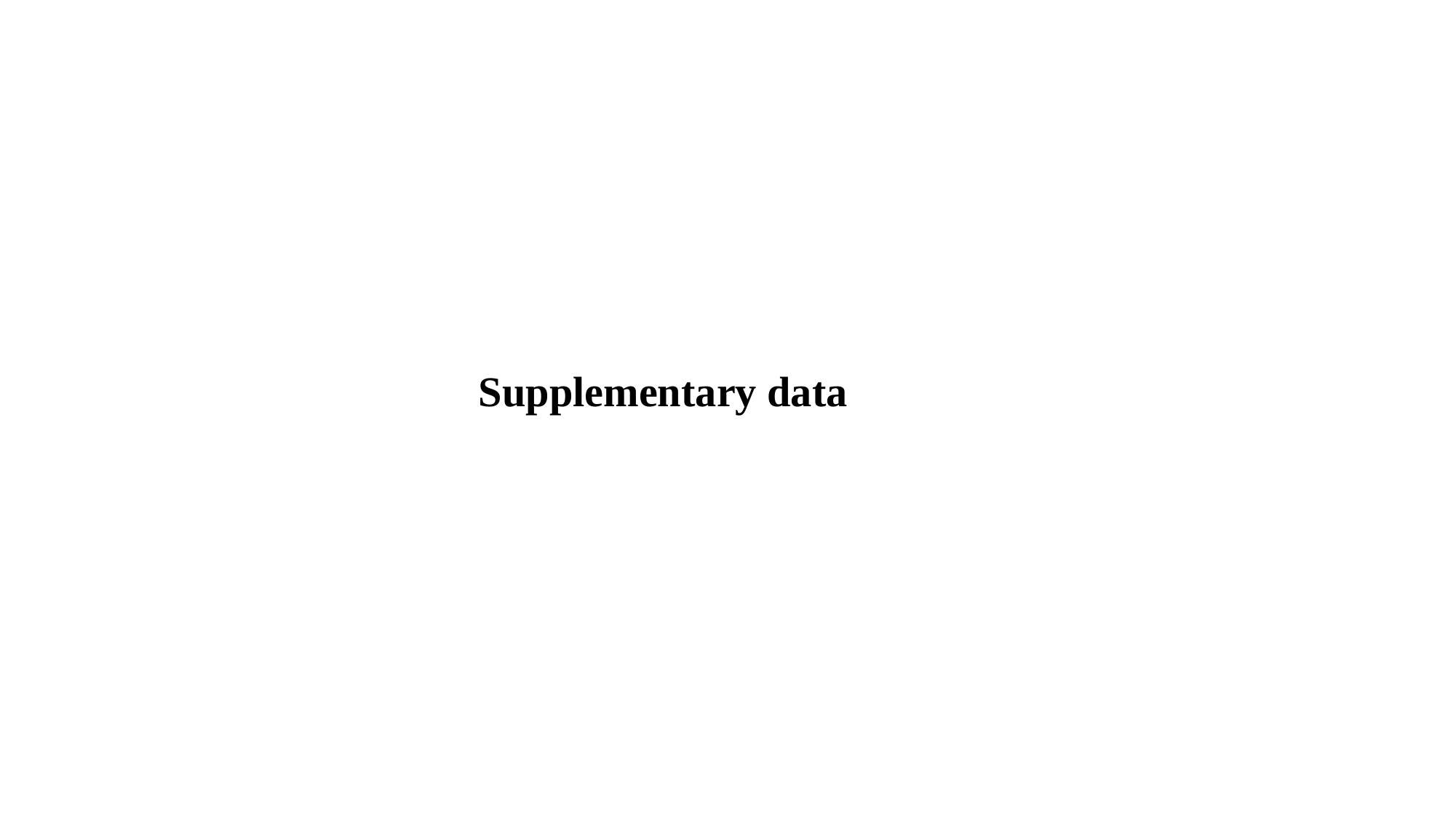

# Supplementary data

## Slide 2
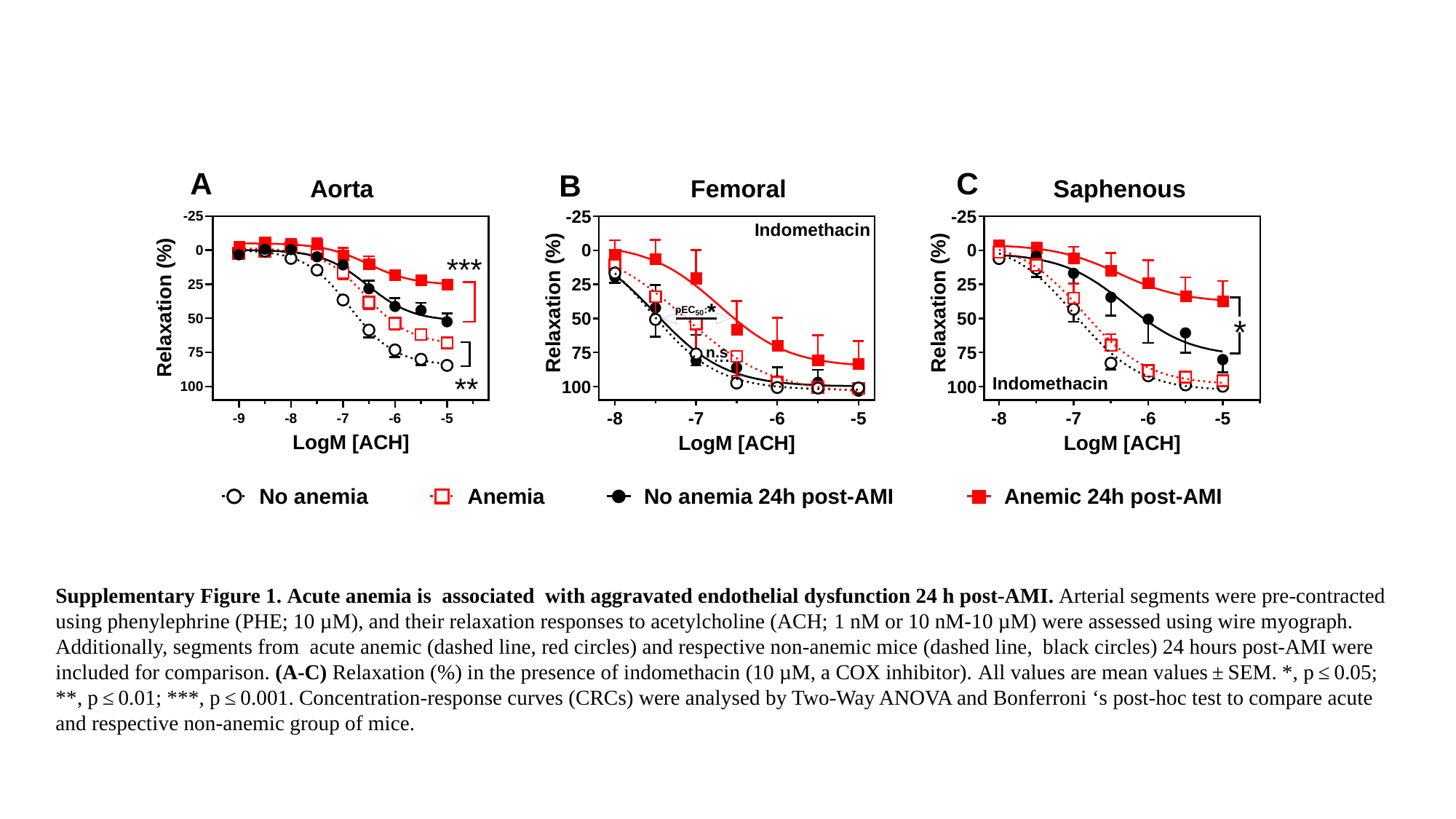

Supplementary Figure 1. Acute anemia is associated with aggravated endothelial dysfunction 24 h post-AMI. Arterial segments were pre-contracted using phenylephrine (PHE; 10 µM), and their relaxation responses to acetylcholine (ACH; 1 nM or 10 nM-10 µM) were assessed using wire myograph. Additionally, segments from acute anemic (dashed line, red circles) and respective non-anemic mice (dashed line, black circles) 24 hours post-AMI were included for comparison. (A-C) Relaxation (%) in the presence of indomethacin (10 µM, a COX inhibitor). All values are mean values ± SEM. *, p ≤ 0.05; **, p ≤ 0.01; ***, p ≤ 0.001. Concentration-response curves (CRCs) were analysed by Two-Way ANOVA and Bonferroni ‘s post-hoc test to compare acute and respective non-anemic group of mice.

## Slide 3
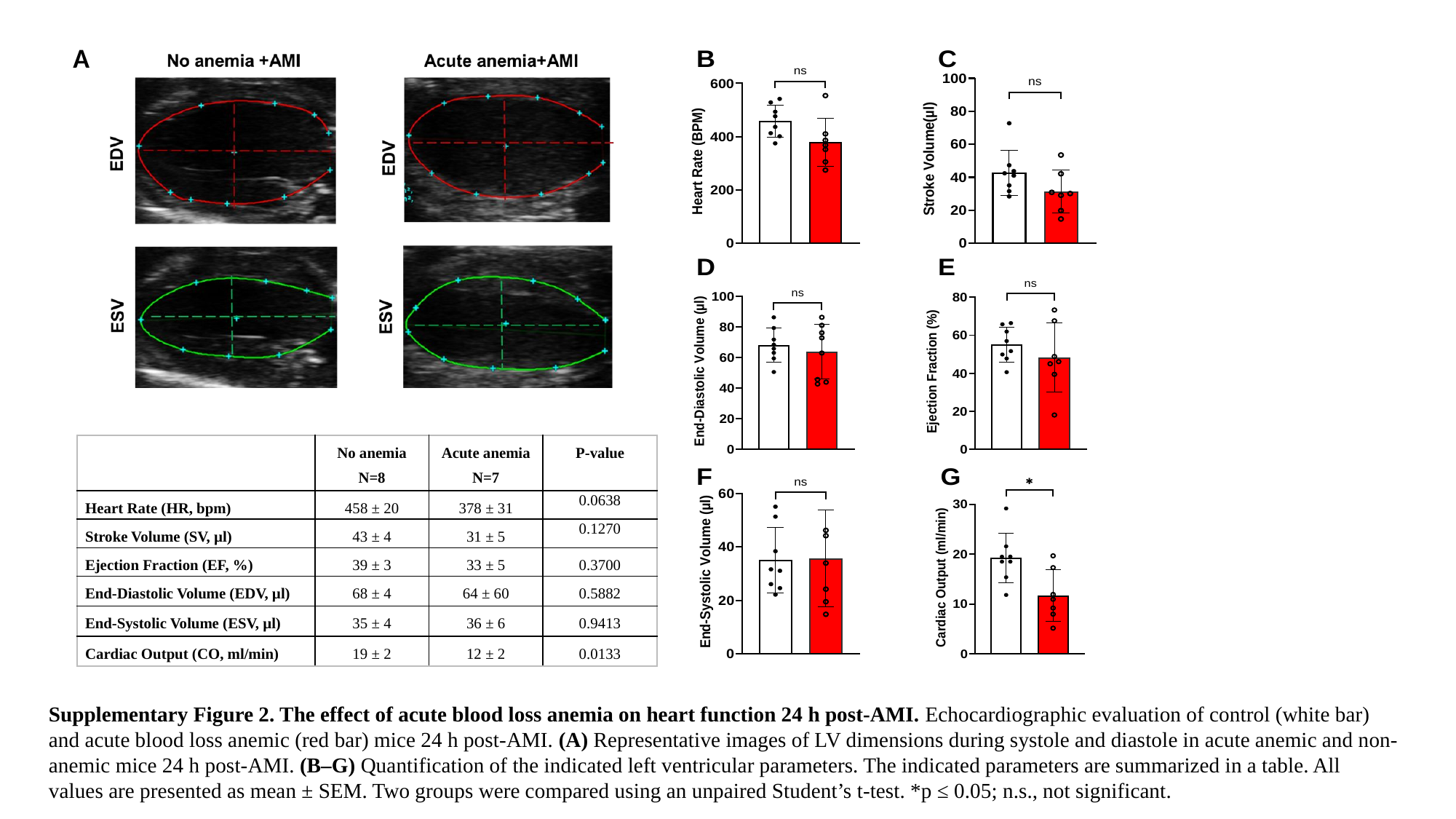

A
| | No anemia N=8 | Acute anemia N=7 | P-value |
| --- | --- | --- | --- |
| Heart Rate (HR, bpm) | 458 ± 20 | 378 ± 31 | 0.0638 |
| Stroke Volume (SV, µl) | 43 ± 4 | 31 ± 5 | 0.1270 |
| Ejection Fraction (EF, %) | 39 ± 3 | 33 ± 5 | 0.3700 |
| End-Diastolic Volume (EDV, µl) | 68 ± 4 | 64 ± 60 | 0.5882 |
| End-Systolic Volume (ESV, µl) | 35 ± 4 | 36 ± 6 | 0.9413 |
| Cardiac Output (CO, ml/min) | 19 ± 2 | 12 ± 2 | 0.0133 |
Supplementary Figure 2. The effect of acute blood loss anemia on heart function 24 h post-AMI. Echocardiographic evaluation of control (white bar) and acute blood loss anemic (red bar) mice 24 h post-AMI. (A) Representative images of LV dimensions during systole and diastole in acute anemic and non-anemic mice 24 h post-AMI. (B–G) Quantification of the indicated left ventricular parameters. The indicated parameters are summarized in a table. All values are presented as mean ± SEM. Two groups were compared using an unpaired Student’s t-test. *p ≤ 0.05; n.s., not significant.

## Slide 4
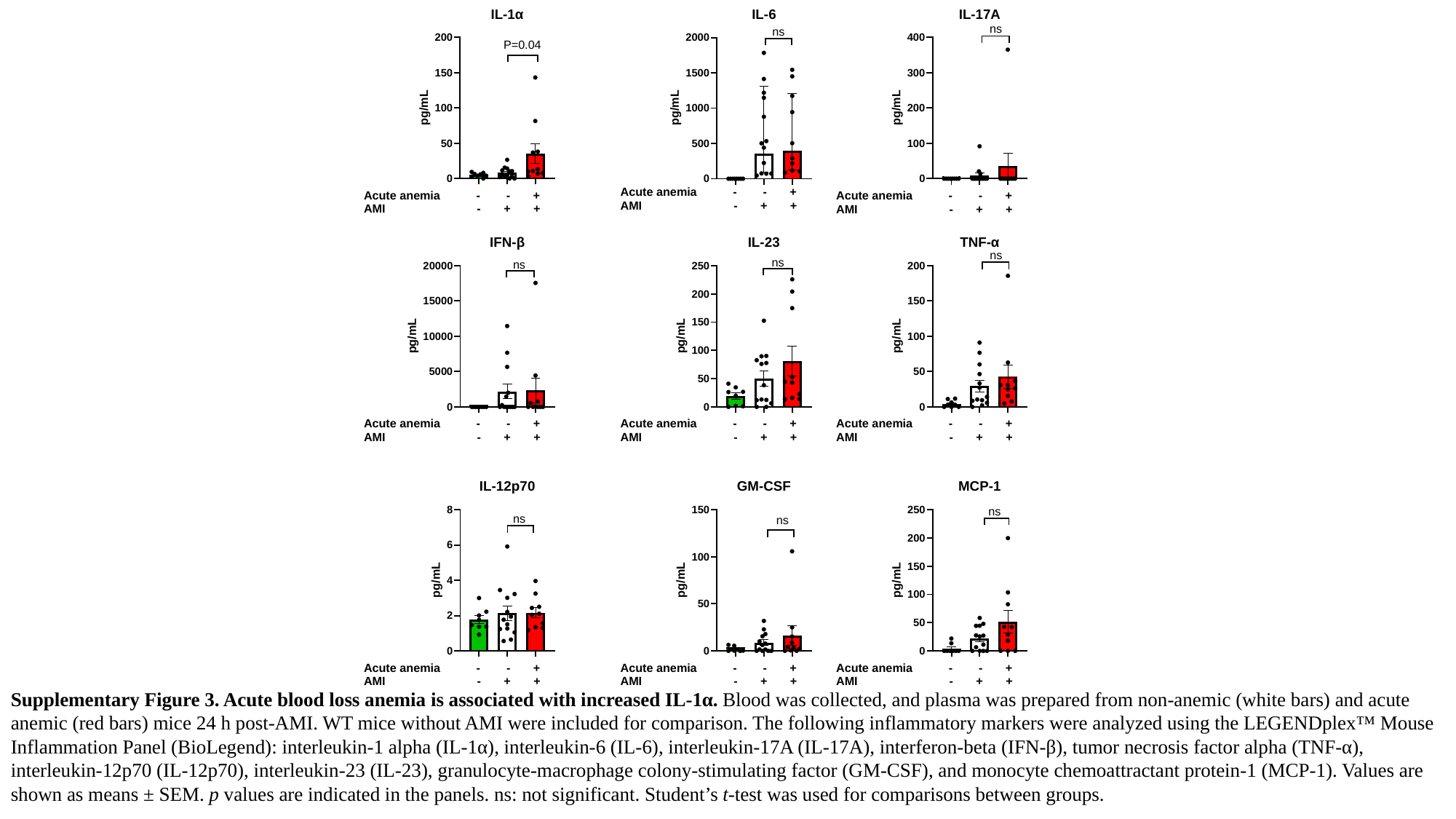

Supplementary Figure 3. Acute blood loss anemia is associated with increased IL-1α. Blood was collected, and plasma was prepared from non-anemic (white bars) and acute anemic (red bars) mice 24 h post-AMI. WT mice without AMI were included for comparison. The following inflammatory markers were analyzed using the LEGENDplex™ Mouse Inflammation Panel (BioLegend): interleukin-1 alpha (IL-1α), interleukin-6 (IL-6), interleukin-17A (IL-17A), interferon-beta (IFN-β), tumor necrosis factor alpha (TNF-α), interleukin-12p70 (IL-12p70), interleukin-23 (IL-23), granulocyte-macrophage colony-stimulating factor (GM-CSF), and monocyte chemoattractant protein-1 (MCP-1). Values are shown as means ± SEM. p values are indicated in the panels. ns: not significant. Student’s t-test was used for comparisons between groups.

## Slide 5
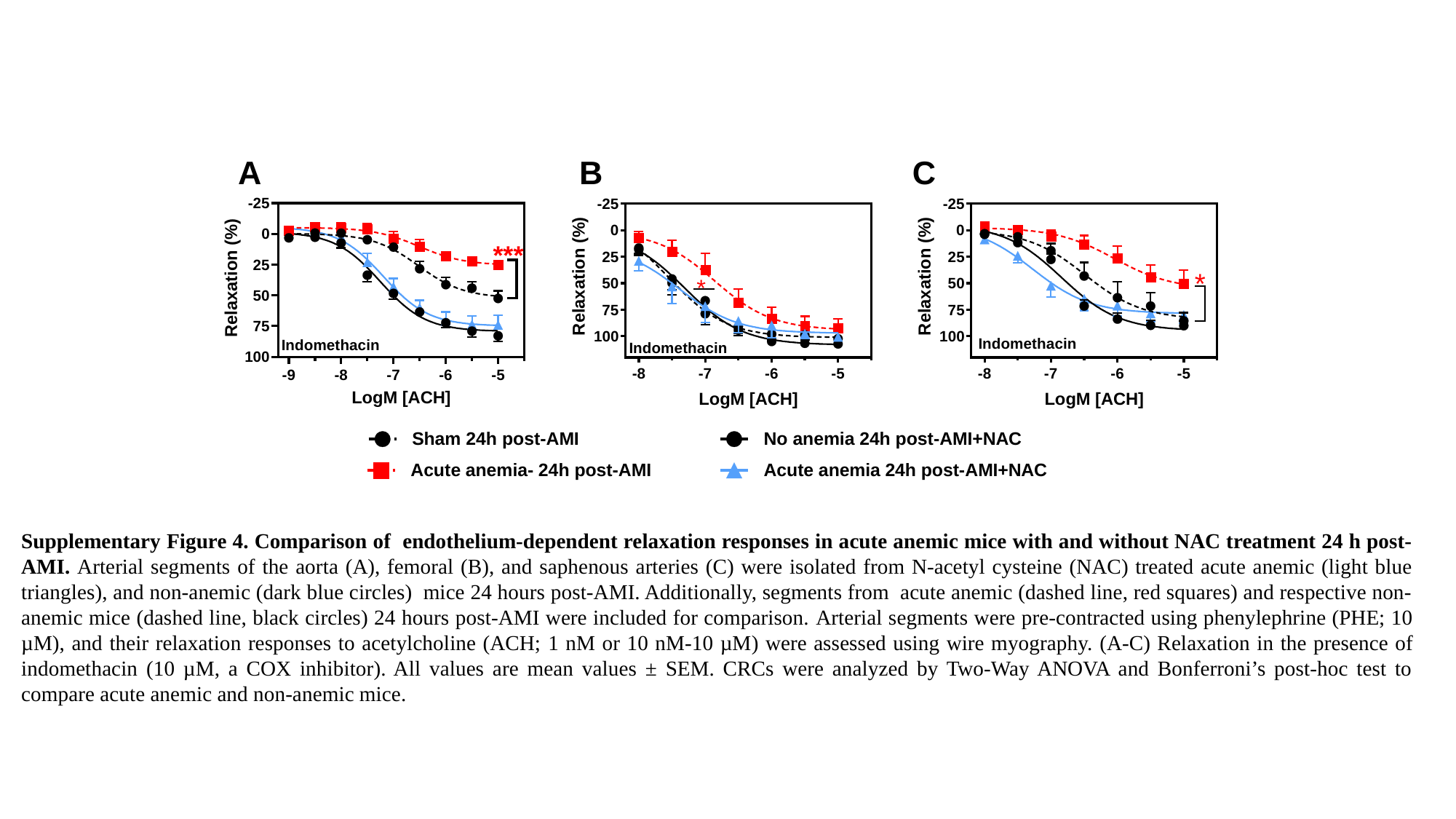

Supplementary Figure 4. Comparison of endothelium-dependent relaxation responses in acute anemic mice with and without NAC treatment 24 h post-AMI. Arterial segments of the aorta (A), femoral (B), and saphenous arteries (C) were isolated from N-acetyl cysteine (NAC) treated acute anemic (light blue triangles), and non-anemic (dark blue circles) mice 24 hours post-AMI. Additionally, segments from acute anemic (dashed line, red squares) and respective non-anemic mice (dashed line, black circles) 24 hours post-AMI were included for comparison. Arterial segments were pre-contracted using phenylephrine (PHE; 10 µM), and their relaxation responses to acetylcholine (ACH; 1 nM or 10 nM-10 µM) were assessed using wire myography. (A-C) Relaxation in the presence of indomethacin (10 µM, a COX inhibitor). All values are mean values ± SEM. CRCs were analyzed by Two-Way ANOVA and Bonferroni’s post-hoc test to compare acute anemic and non-anemic mice.

## Slide 6
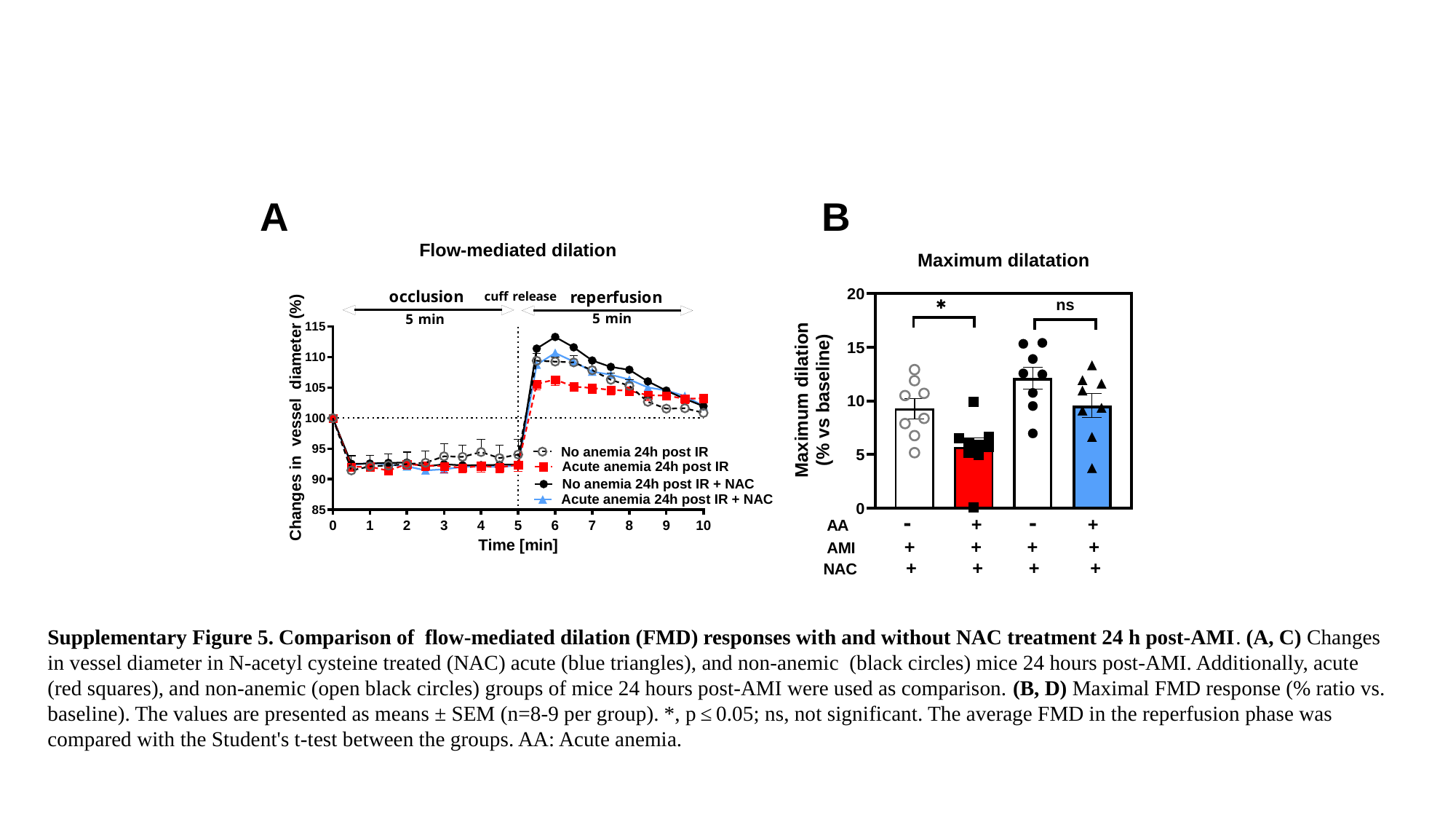

Supplementary Figure 5. Comparison of flow-mediated dilation (FMD) responses with and without NAC treatment 24 h post-AMI. (A, C) Changes in vessel diameter in N-acetyl cysteine treated (NAC) acute (blue triangles), and non-anemic (black circles) mice 24 hours post-AMI. Additionally, acute (red squares), and non-anemic (open black circles) groups of mice 24 hours post-AMI were used as comparison. (B, D) Maximal FMD response (% ratio vs. baseline). The values are presented as means ± SEM (n=8-9 per group). *, p ≤ 0.05; ns, not significant. The average FMD in the reperfusion phase was compared with the Student's t-test between the groups. AA: Acute anemia.

## Slide 7
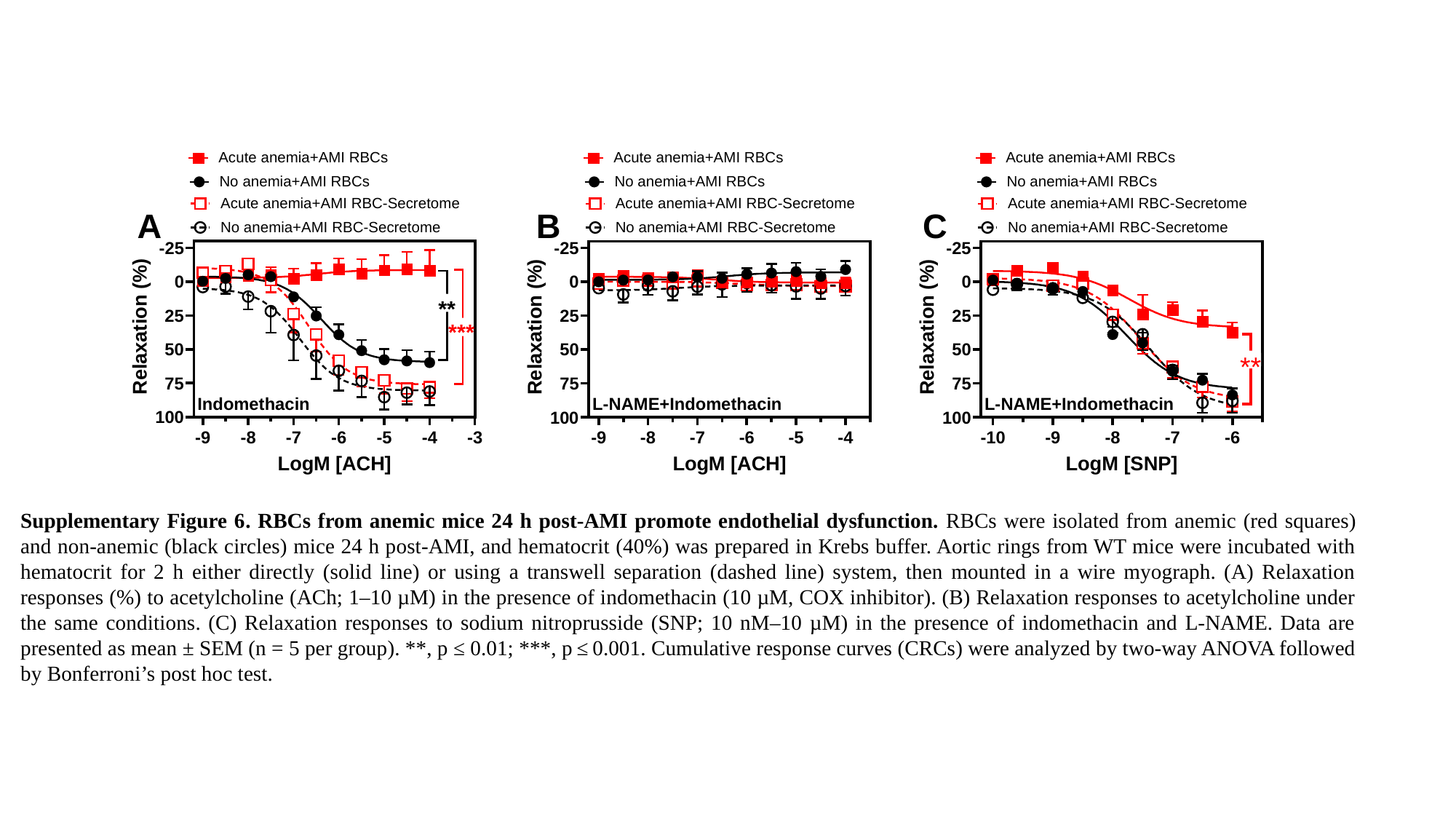

Supplementary Figure 6. RBCs from anemic mice 24 h post-AMI promote endothelial dysfunction. RBCs were isolated from anemic (red squares) and non-anemic (black circles) mice 24 h post-AMI, and hematocrit (40%) was prepared in Krebs buffer. Aortic rings from WT mice were incubated with hematocrit for 2 h either directly (solid line) or using a transwell separation (dashed line) system, then mounted in a wire myograph. (A) Relaxation responses (%) to acetylcholine (ACh; 1–10 µM) in the presence of indomethacin (10 µM, COX inhibitor). (B) Relaxation responses to acetylcholine under the same conditions. (C) Relaxation responses to sodium nitroprusside (SNP; 10 nM–10 µM) in the presence of indomethacin and L-NAME. Data are presented as mean ± SEM (n = 5 per group). **, p ≤ 0.01; ***, p ≤ 0.001. Cumulative response curves (CRCs) were analyzed by two-way ANOVA followed by Bonferroni’s post hoc test.

## Slide 8
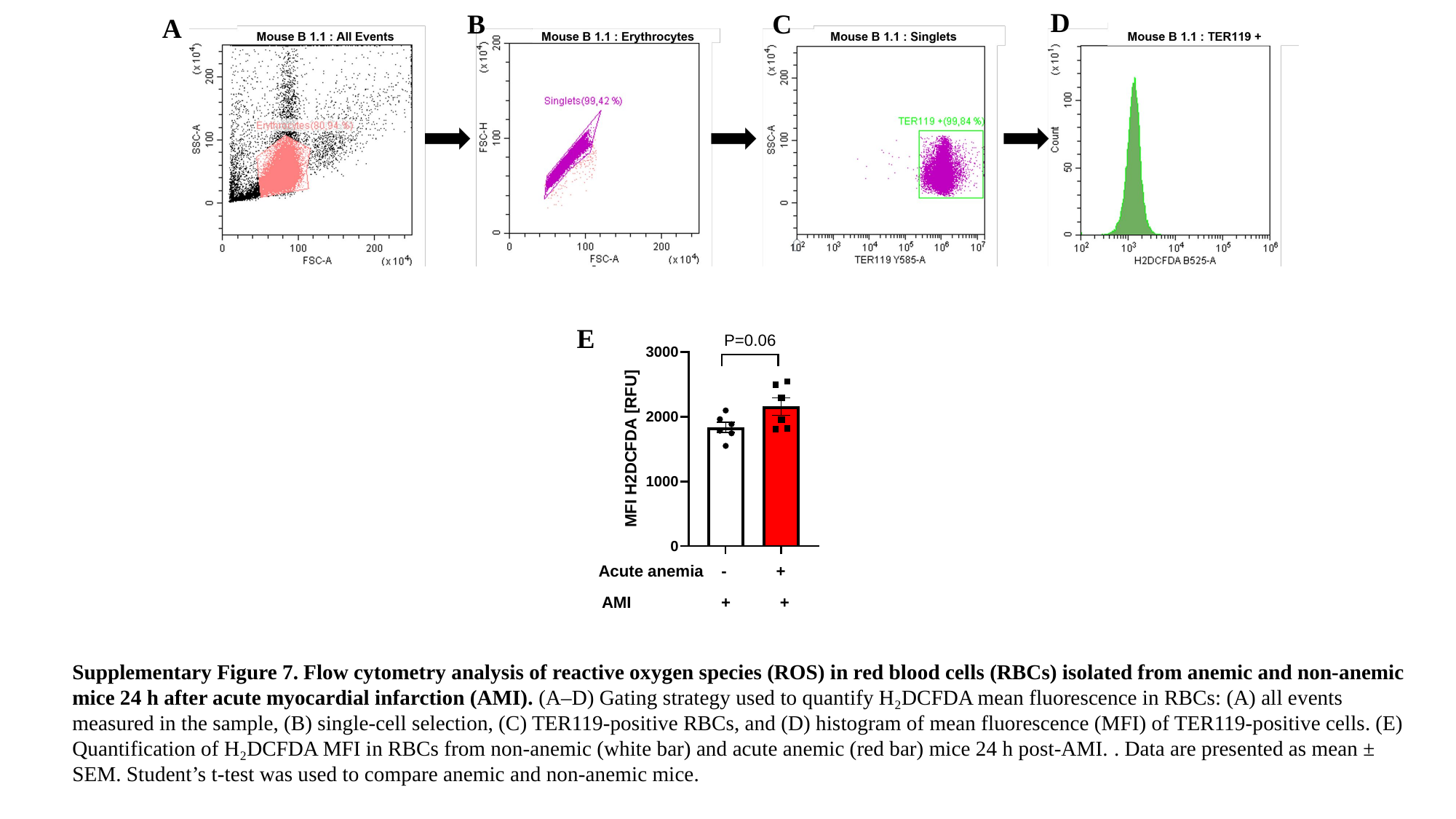

D
B
C
A
E
Supplementary Figure 7. Flow cytometry analysis of reactive oxygen species (ROS) in red blood cells (RBCs) isolated from anemic and non-anemic mice 24 h after acute myocardial infarction (AMI). (A–D) Gating strategy used to quantify H₂DCFDA mean fluorescence in RBCs: (A) all events measured in the sample, (B) single-cell selection, (C) TER119-positive RBCs, and (D) histogram of mean fluorescence (MFI) of TER119-positive cells. (E) Quantification of H₂DCFDA MFI in RBCs from non-anemic (white bar) and acute anemic (red bar) mice 24 h post-AMI. . Data are presented as mean ± SEM. Student’s t-test was used to compare anemic and non-anemic mice.

## Slide 9
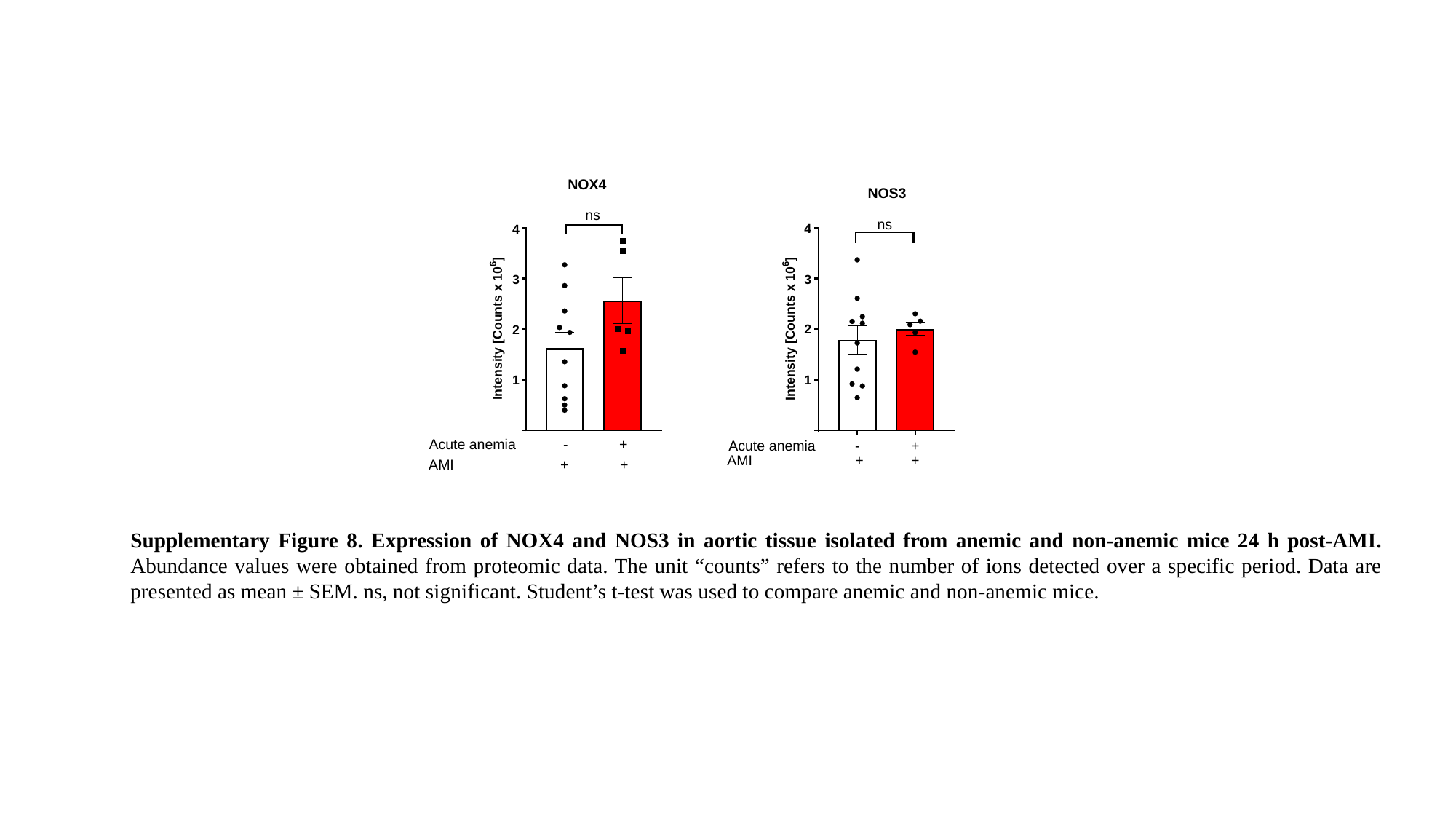

Supplementary Figure 8. Expression of NOX4 and NOS3 in aortic tissue isolated from anemic and non-anemic mice 24 h post-AMI. Abundance values were obtained from proteomic data. The unit “counts” refers to the number of ions detected over a specific period. Data are presented as mean ± SEM. ns, not significant. Student’s t-test was used to compare anemic and non-anemic mice.

## Slide 10
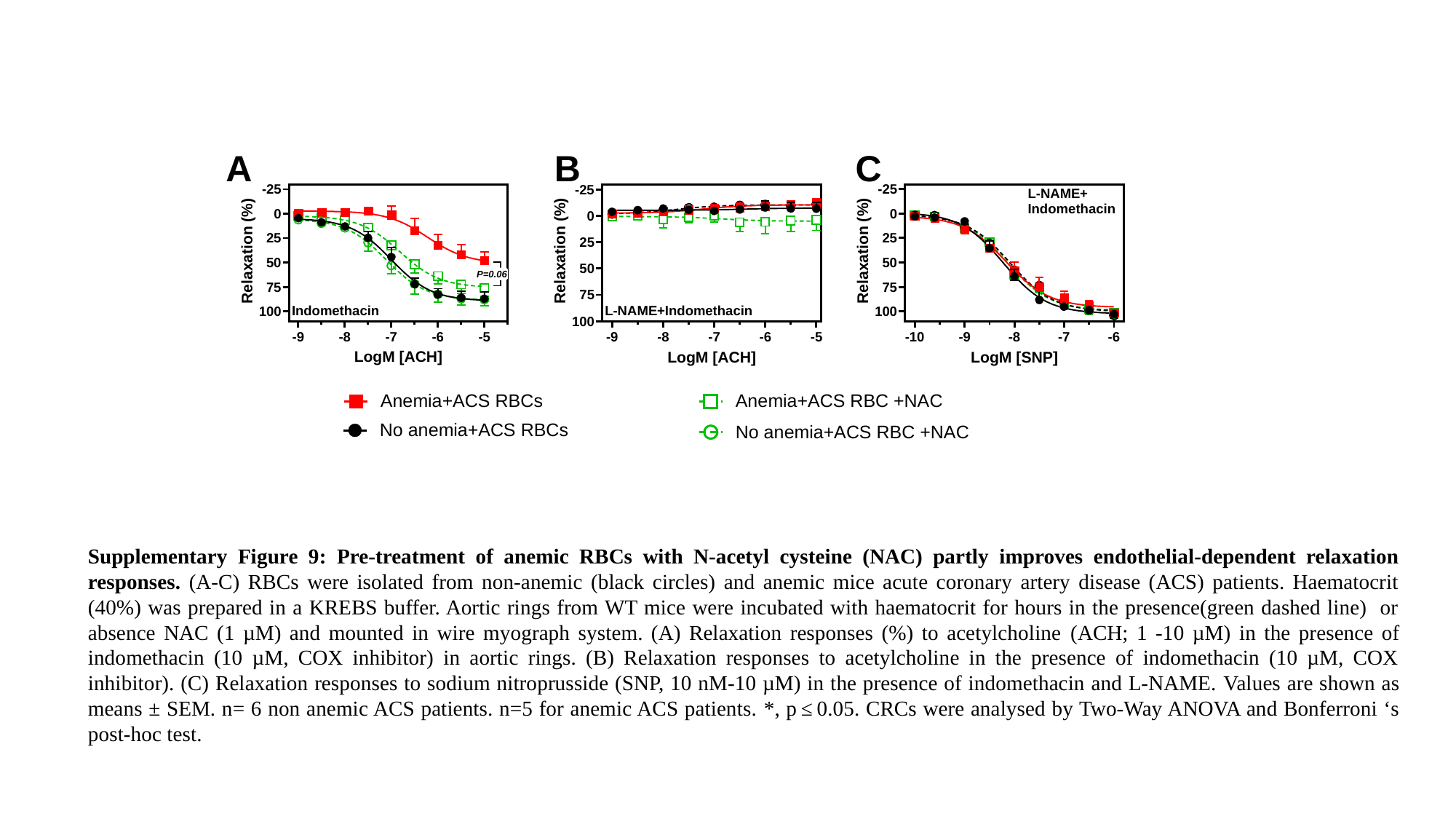

Supplementary Figure 9: Pre-treatment of anemic RBCs with N-acetyl cysteine (NAC) partly improves endothelial-dependent relaxation responses. (A-C) RBCs were isolated from non-anemic (black circles) and anemic mice acute coronary artery disease (ACS) patients. Haematocrit (40%) was prepared in a KREBS buffer. Aortic rings from WT mice were incubated with haematocrit for hours in the presence(green dashed line) or absence NAC (1 µM) and mounted in wire myograph system. (A) Relaxation responses (%) to acetylcholine (ACH; 1 -10 µM) in the presence of indomethacin (10 µM, COX inhibitor) in aortic rings. (B) Relaxation responses to acetylcholine in the presence of indomethacin (10 µM, COX inhibitor). (C) Relaxation responses to sodium nitroprusside (SNP, 10 nM-10 µM) in the presence of indomethacin and L-NAME. Values are shown as means ± SEM. n= 6 non anemic ACS patients. n=5 for anemic ACS patients. *, p ≤ 0.05. CRCs were analysed by Two-Way ANOVA and Bonferroni ‘s post-hoc test.

## Slide 11
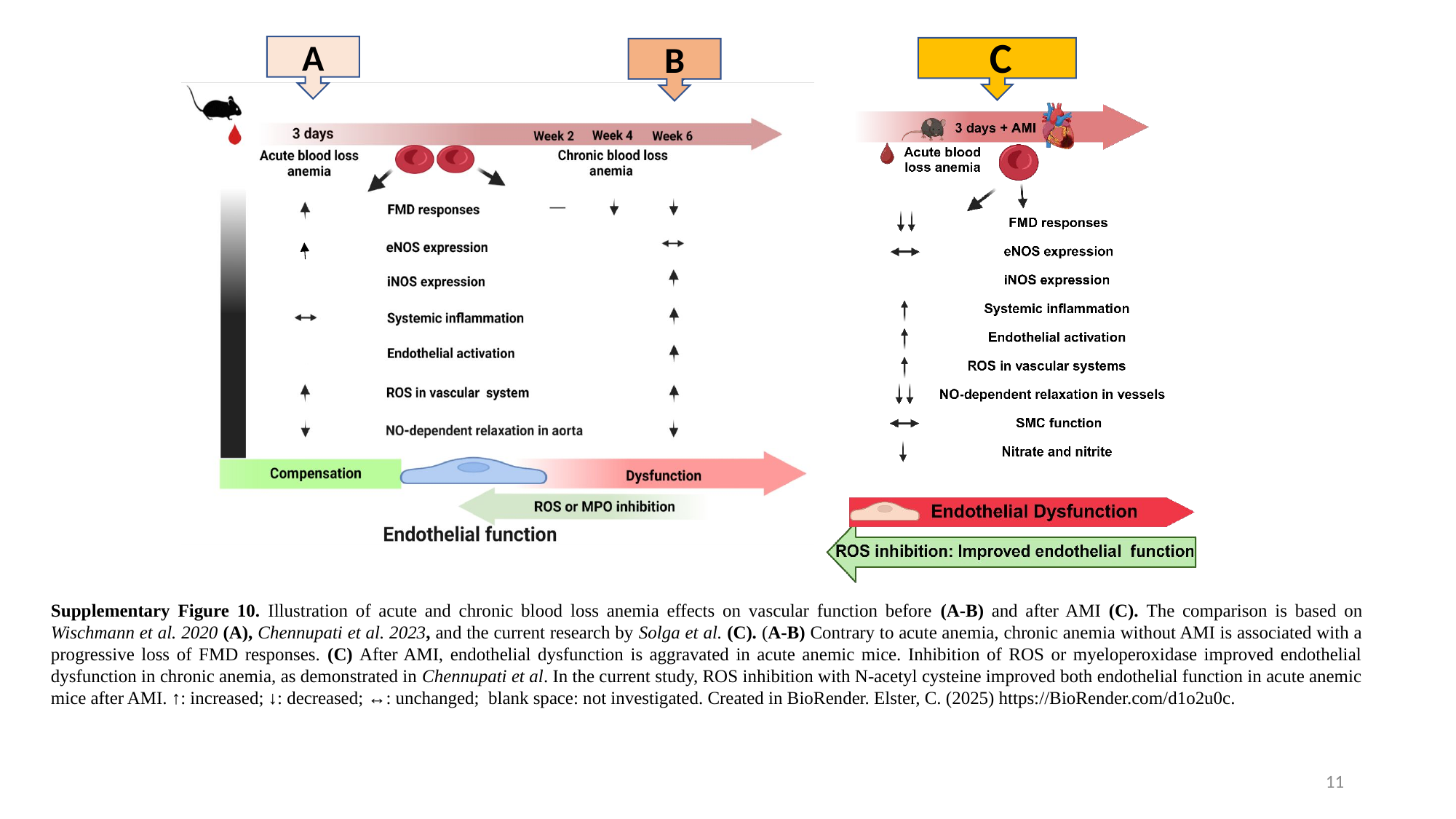

A
C
B
A
B
Supplementary Figure 10. Illustration of acute and chronic blood loss anemia effects on vascular function before (A-B) and after AMI (C). The comparison is based on Wischmann et al. 2020 (A), Chennupati et al. 2023, and the current research by Solga et al. (C). (A-B) Contrary to acute anemia, chronic anemia without AMI is associated with a progressive loss of FMD responses. (C) After AMI, endothelial dysfunction is aggravated in acute anemic mice. Inhibition of ROS or myeloperoxidase improved endothelial dysfunction in chronic anemia, as demonstrated in Chennupati et al. In the current study, ROS inhibition with N-acetyl cysteine improved both endothelial function in acute anemic mice after AMI. ↑: increased; ↓: decreased; ↔: unchanged; blank space: not investigated. Created in BioRender. Elster, C. (2025) https://BioRender.com/d1o2u0c.
11

## Slide 12
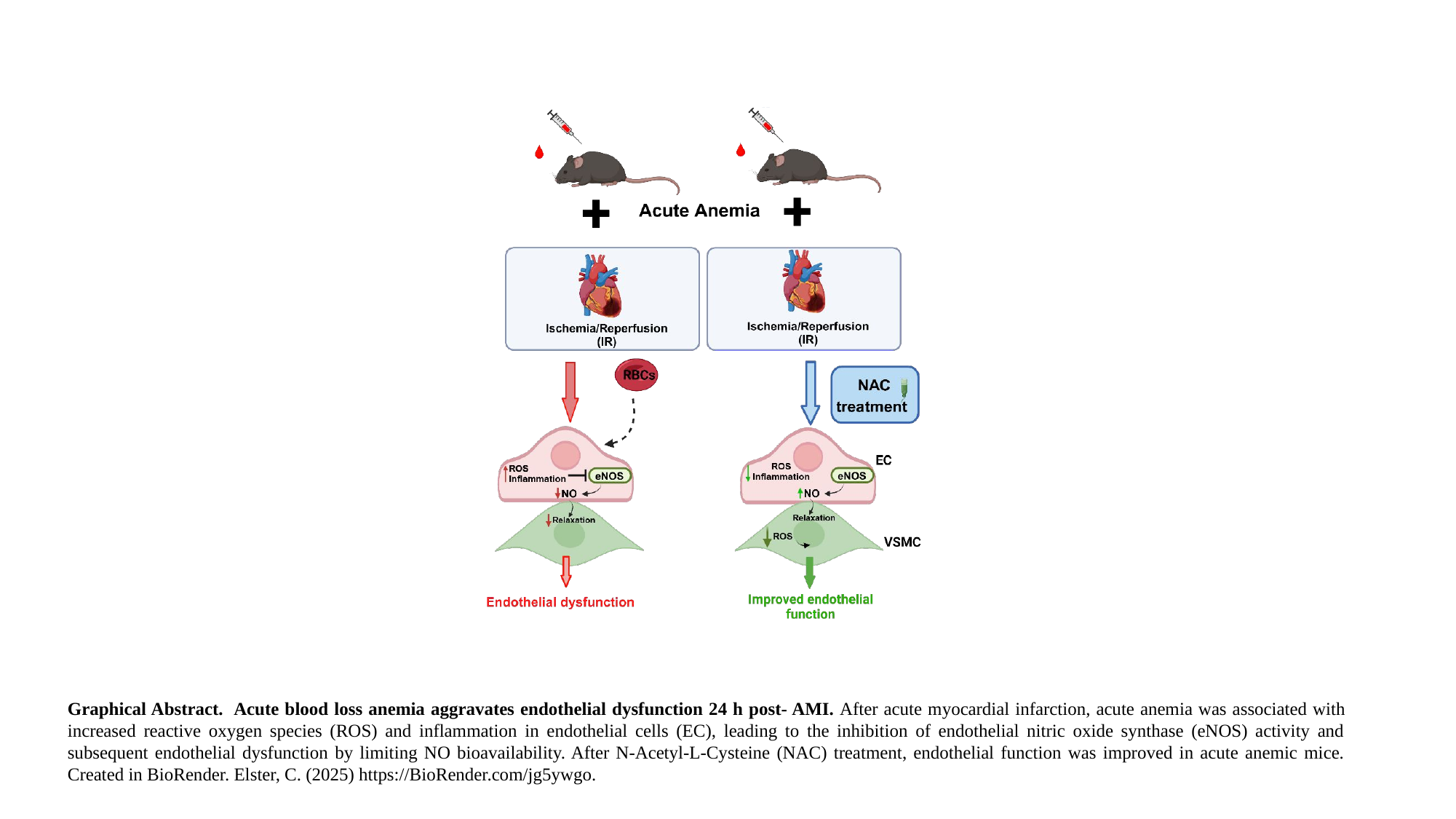

Graphical Abstract. Acute blood loss anemia aggravates endothelial dysfunction 24 h post- AMI. After acute myocardial infarction, acute anemia was associated with increased reactive oxygen species (ROS) and inflammation in endothelial cells (EC), leading to the inhibition of endothelial nitric oxide synthase (eNOS) activity and subsequent endothelial dysfunction by limiting NO bioavailability. After N-Acetyl-L-Cysteine (NAC) treatment, endothelial function was improved in acute anemic mice. Created in BioRender. Elster, C. (2025) https://BioRender.com/jg5ywgo.
