# Supplementary material for: Acute blood loss anemia aggravates endothelial dysfunction after acute myocardial infarction
Source: Front Cardiovasc Med. 2025 Oct 13;12:1635293. doi: 10.3389/fcvm.2025.1635293 (PMC12554764; doi:10.3389/fcvm.2025.1635293)
Supplement: Supplementary file 2 [file Presentation2.pptx]

## Slide 1
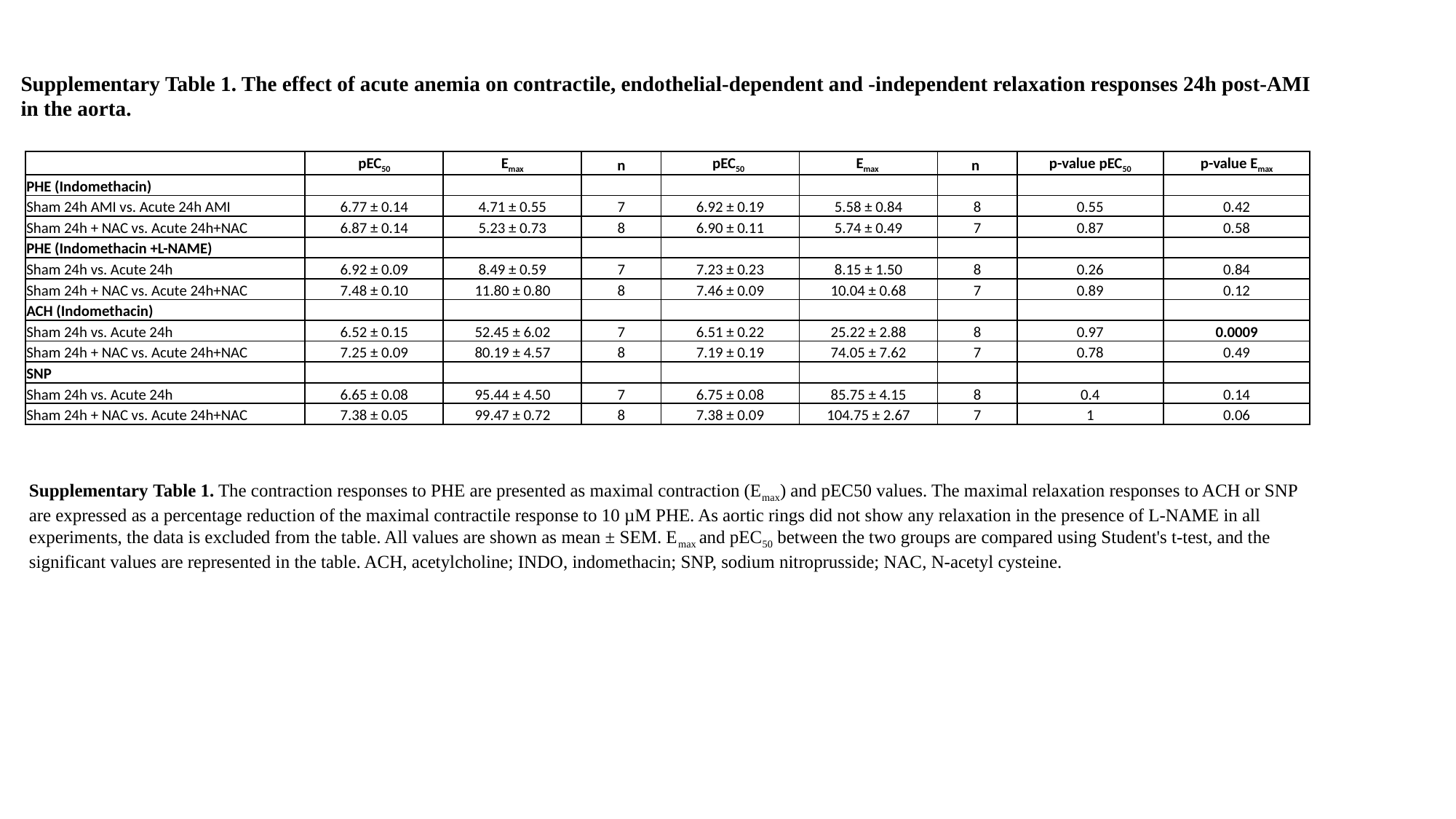

Supplementary Table 1. The effect of acute anemia on contractile, endothelial-dependent and -independent relaxation responses 24h post-AMI in the aorta.
| | pEC50 | Emax | n | pEC50 | Emax | n | p-value pEC50 | p-value Emax |
| --- | --- | --- | --- | --- | --- | --- | --- | --- |
| PHE (Indomethacin) | | | | | | | | |
| Sham 24h AMI vs. Acute 24h AMI | 6.77 ± 0.14 | 4.71 ± 0.55 | 7 | 6.92 ± 0.19 | 5.58 ± 0.84 | 8 | 0.55 | 0.42 |
| Sham 24h + NAC vs. Acute 24h+NAC | 6.87 ± 0.14 | 5.23 ± 0.73 | 8 | 6.90 ± 0.11 | 5.74 ± 0.49 | 7 | 0.87 | 0.58 |
| PHE (Indomethacin +L-NAME) | | | | | | | | |
| Sham 24h vs. Acute 24h | 6.92 ± 0.09 | 8.49 ± 0.59 | 7 | 7.23 ± 0.23 | 8.15 ± 1.50 | 8 | 0.26 | 0.84 |
| Sham 24h + NAC vs. Acute 24h+NAC | 7.48 ± 0.10 | 11.80 ± 0.80 | 8 | 7.46 ± 0.09 | 10.04 ± 0.68 | 7 | 0.89 | 0.12 |
| ACH (Indomethacin) | | | | | | | | |
| Sham 24h vs. Acute 24h | 6.52 ± 0.15 | 52.45 ± 6.02 | 7 | 6.51 ± 0.22 | 25.22 ± 2.88 | 8 | 0.97 | 0.0009 |
| Sham 24h + NAC vs. Acute 24h+NAC | 7.25 ± 0.09 | 80.19 ± 4.57 | 8 | 7.19 ± 0.19 | 74.05 ± 7.62 | 7 | 0.78 | 0.49 |
| SNP | | | | | | | | |
| Sham 24h vs. Acute 24h | 6.65 ± 0.08 | 95.44 ± 4.50 | 7 | 6.75 ± 0.08 | 85.75 ± 4.15 | 8 | 0.4 | 0.14 |
| Sham 24h + NAC vs. Acute 24h+NAC | 7.38 ± 0.05 | 99.47 ± 0.72 | 8 | 7.38 ± 0.09 | 104.75 ± 2.67 | 7 | 1 | 0.06 |
Supplementary Table 1. The contraction responses to PHE are presented as maximal contraction (Emax) and pEC50 values. The maximal relaxation responses to ACH or SNP are expressed as a percentage reduction of the maximal contractile response to 10 µM PHE. As aortic rings did not show any relaxation in the presence of L-NAME in all experiments, the data is excluded from the table. All values are shown as mean ± SEM. Emax and pEC50 between the two groups are compared using Student's t-test, and the significant values are represented in the table. ACH, acetylcholine; INDO, indomethacin; SNP, sodium nitroprusside; NAC, N-acetyl cysteine.

## Slide 2
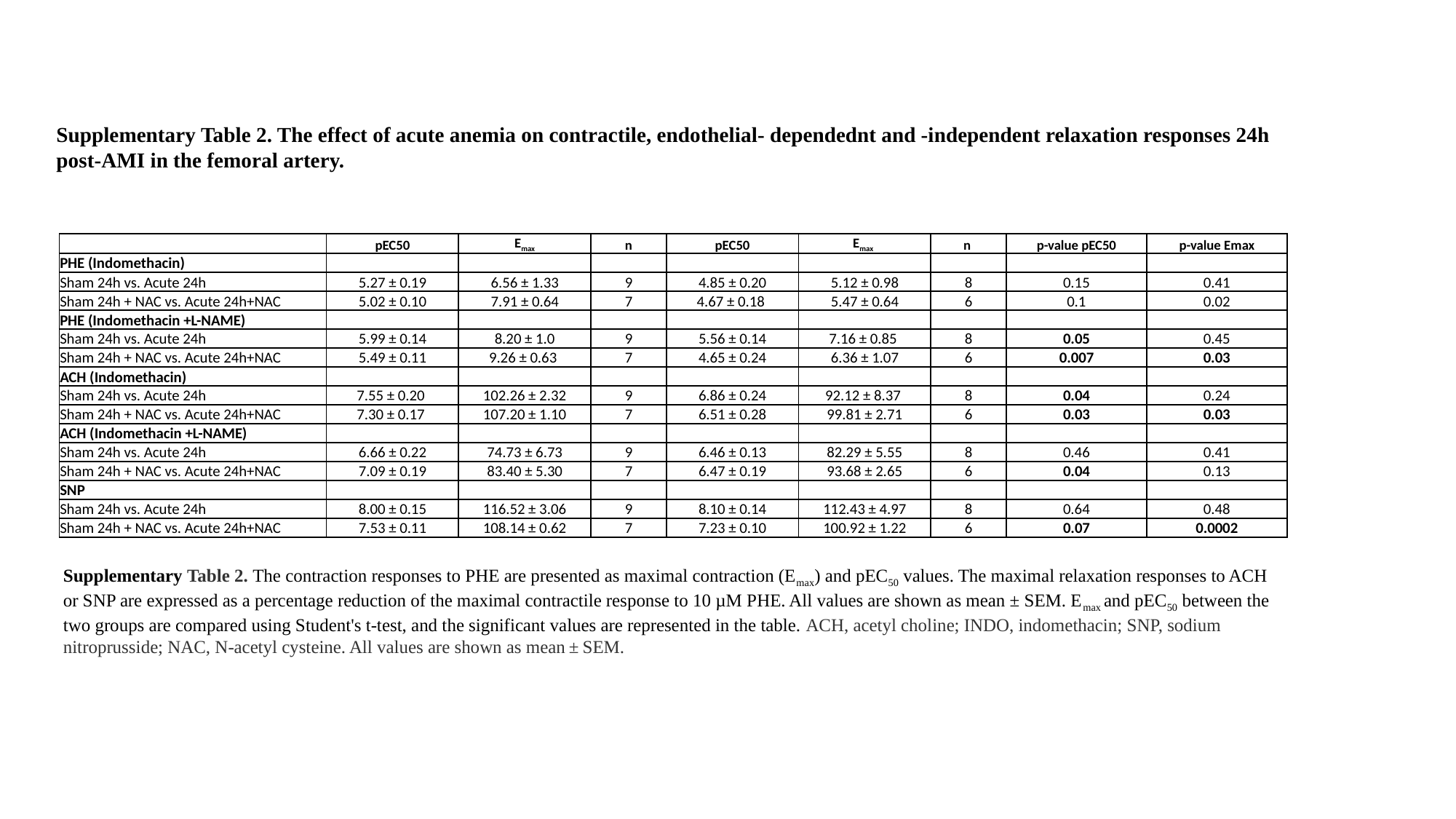

Supplementary Table 2. The effect of acute anemia on contractile, endothelial- dependednt and -independent relaxation responses 24h post-AMI in the femoral artery.
| | pEC50 | Emax | n | pEC50 | Emax | n | p-value pEC50 | p-value Emax |
| --- | --- | --- | --- | --- | --- | --- | --- | --- |
| PHE (Indomethacin) | | | | | | | | |
| Sham 24h vs. Acute 24h | 5.27 ± 0.19 | 6.56 ± 1.33 | 9 | 4.85 ± 0.20 | 5.12 ± 0.98 | 8 | 0.15 | 0.41 |
| Sham 24h + NAC vs. Acute 24h+NAC | 5.02 ± 0.10 | 7.91 ± 0.64 | 7 | 4.67 ± 0.18 | 5.47 ± 0.64 | 6 | 0.1 | 0.02 |
| PHE (Indomethacin +L-NAME) | | | | | | | | |
| Sham 24h vs. Acute 24h | 5.99 ± 0.14 | 8.20 ± 1.0 | 9 | 5.56 ± 0.14 | 7.16 ± 0.85 | 8 | 0.05 | 0.45 |
| Sham 24h + NAC vs. Acute 24h+NAC | 5.49 ± 0.11 | 9.26 ± 0.63 | 7 | 4.65 ± 0.24 | 6.36 ± 1.07 | 6 | 0.007 | 0.03 |
| ACH (Indomethacin) | | | | | | | | |
| Sham 24h vs. Acute 24h | 7.55 ± 0.20 | 102.26 ± 2.32 | 9 | 6.86 ± 0.24 | 92.12 ± 8.37 | 8 | 0.04 | 0.24 |
| Sham 24h + NAC vs. Acute 24h+NAC | 7.30 ± 0.17 | 107.20 ± 1.10 | 7 | 6.51 ± 0.28 | 99.81 ± 2.71 | 6 | 0.03 | 0.03 |
| ACH (Indomethacin +L-NAME) | | | | | | | | |
| Sham 24h vs. Acute 24h | 6.66 ± 0.22 | 74.73 ± 6.73 | 9 | 6.46 ± 0.13 | 82.29 ± 5.55 | 8 | 0.46 | 0.41 |
| Sham 24h + NAC vs. Acute 24h+NAC | 7.09 ± 0.19 | 83.40 ± 5.30 | 7 | 6.47 ± 0.19 | 93.68 ± 2.65 | 6 | 0.04 | 0.13 |
| SNP | | | | | | | | |
| Sham 24h vs. Acute 24h | 8.00 ± 0.15 | 116.52 ± 3.06 | 9 | 8.10 ± 0.14 | 112.43 ± 4.97 | 8 | 0.64 | 0.48 |
| Sham 24h + NAC vs. Acute 24h+NAC | 7.53 ± 0.11 | 108.14 ± 0.62 | 7 | 7.23 ± 0.10 | 100.92 ± 1.22 | 6 | 0.07 | 0.0002 |
Supplementary Table 2. The contraction responses to PHE are presented as maximal contraction (Emax) and pEC50 values. The maximal relaxation responses to ACH or SNP are expressed as a percentage reduction of the maximal contractile response to 10 µM PHE. All values are shown as mean ± SEM. Emax and pEC50 between the two groups are compared using Student's t-test, and the significant values are represented in the table. ACH, acetyl choline; INDO, indomethacin; SNP, sodium nitroprusside; NAC, N-acetyl cysteine. All values are shown as mean ± SEM.

## Slide 3
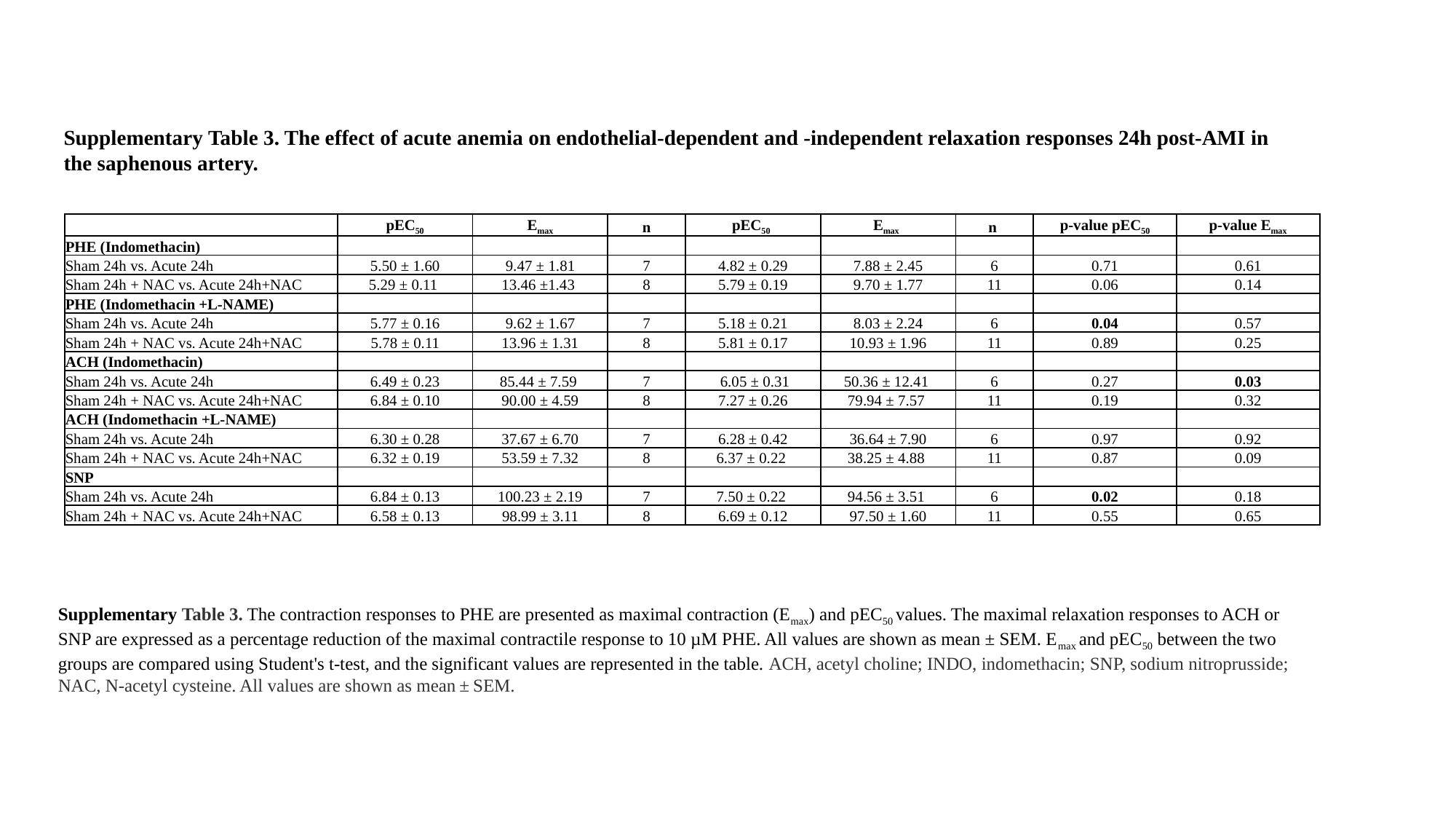

Supplementary Table 3. The effect of acute anemia on endothelial-dependent and -independent relaxation responses 24h post-AMI in the saphenous artery.
| | pEC50 | Emax | n | pEC50 | Emax | n | p-value pEC50 | p-value Emax |
| --- | --- | --- | --- | --- | --- | --- | --- | --- |
| PHE (Indomethacin) | | | | | | | | |
| Sham 24h vs. Acute 24h | 5.50 ± 1.60 | 9.47 ± 1.81 | 7 | 4.82 ± 0.29 | 7.88 ± 2.45 | 6 | 0.71 | 0.61 |
| Sham 24h + NAC vs. Acute 24h+NAC | 5.29 ± 0.11 | 13.46 ±1.43 | 8 | 5.79 ± 0.19 | 9.70 ± 1.77 | 11 | 0.06 | 0.14 |
| PHE (Indomethacin +L-NAME) | | | | | | | | |
| Sham 24h vs. Acute 24h | 5.77 ± 0.16 | 9.62 ± 1.67 | 7 | 5.18 ± 0.21 | 8.03 ± 2.24 | 6 | 0.04 | 0.57 |
| Sham 24h + NAC vs. Acute 24h+NAC | 5.78 ± 0.11 | 13.96 ± 1.31 | 8 | 5.81 ± 0.17 | 10.93 ± 1.96 | 11 | 0.89 | 0.25 |
| ACH (Indomethacin) | | | | | | | | |
| Sham 24h vs. Acute 24h | 6.49 ± 0.23 | 85.44 ± 7.59 | 7 | 6.05 ± 0.31 | 50.36 ± 12.41 | 6 | 0.27 | 0.03 |
| Sham 24h + NAC vs. Acute 24h+NAC | 6.84 ± 0.10 | 90.00 ± 4.59 | 8 | 7.27 ± 0.26 | 79.94 ± 7.57 | 11 | 0.19 | 0.32 |
| ACH (Indomethacin +L-NAME) | | | | | | | | |
| Sham 24h vs. Acute 24h | 6.30 ± 0.28 | 37.67 ± 6.70 | 7 | 6.28 ± 0.42 | 36.64 ± 7.90 | 6 | 0.97 | 0.92 |
| Sham 24h + NAC vs. Acute 24h+NAC | 6.32 ± 0.19 | 53.59 ± 7.32 | 8 | 6.37 ± 0.22 | 38.25 ± 4.88 | 11 | 0.87 | 0.09 |
| SNP | | | | | | | | |
| Sham 24h vs. Acute 24h | 6.84 ± 0.13 | 100.23 ± 2.19 | 7 | 7.50 ± 0.22 | 94.56 ± 3.51 | 6 | 0.02 | 0.18 |
| Sham 24h + NAC vs. Acute 24h+NAC | 6.58 ± 0.13 | 98.99 ± 3.11 | 8 | 6.69 ± 0.12 | 97.50 ± 1.60 | 11 | 0.55 | 0.65 |
Supplementary Table 3. The contraction responses to PHE are presented as maximal contraction (Emax) and pEC50 values. The maximal relaxation responses to ACH or SNP are expressed as a percentage reduction of the maximal contractile response to 10 µM PHE. All values are shown as mean ± SEM. Emax and pEC50 between the two groups are compared using Student's t-test, and the significant values are represented in the table. ACH, acetyl choline; INDO, indomethacin; SNP, sodium nitroprusside; NAC, N-acetyl cysteine. All values are shown as mean ± SEM.

## Slide 4
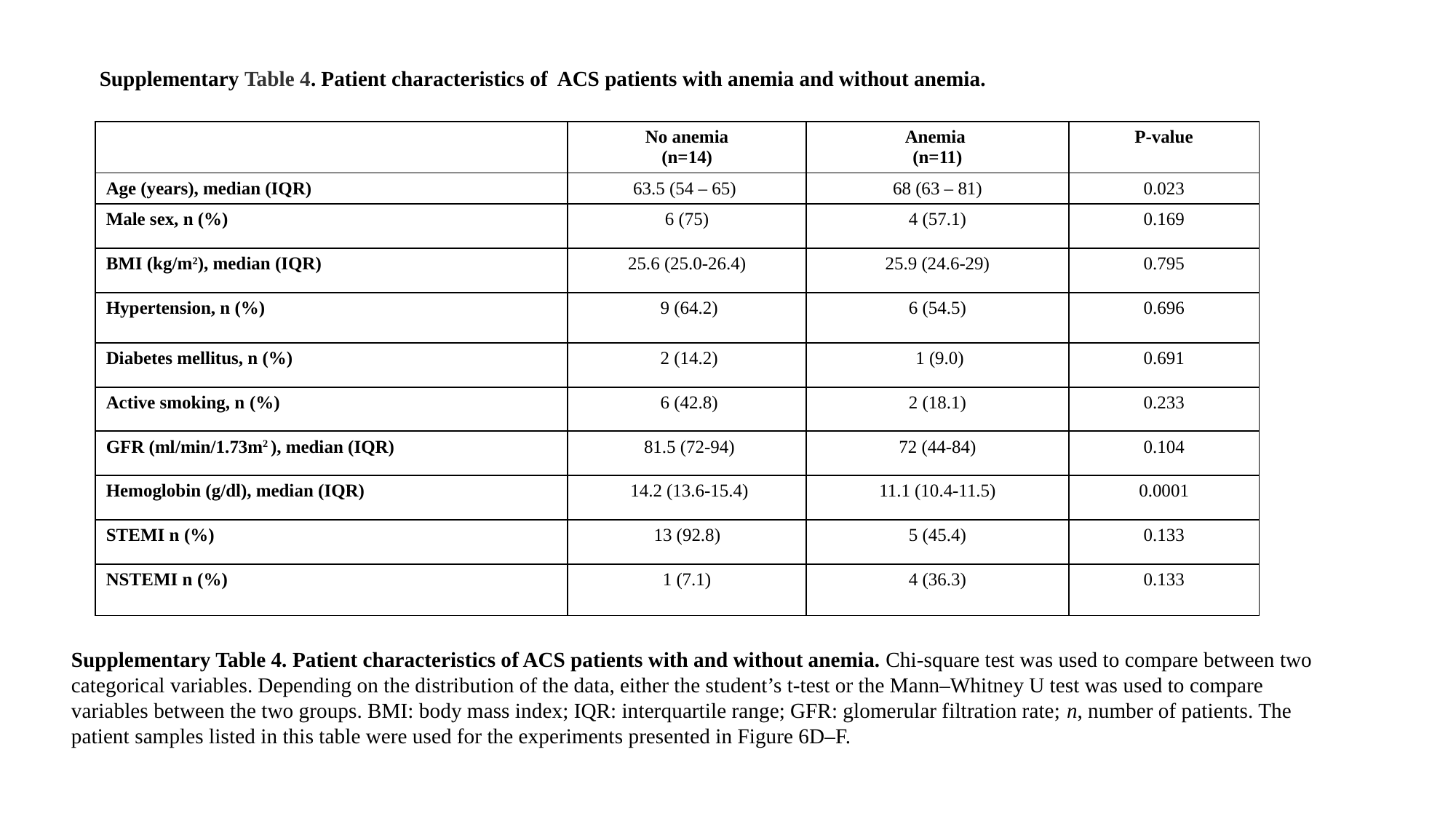

Supplementary Table 4. Patient characteristics of ACS patients with anemia and without anemia.
| | No anemia (n=14) | Anemia (n=11) | P-value |
| --- | --- | --- | --- |
| Age (years), median (IQR) | 63.5 (54 – 65) | 68 (63 – 81) | 0.023 |
| Male sex, n (%) | 6 (75) | 4 (57.1) | 0.169 |
| BMI (kg/m2), median (IQR) | 25.6 (25.0-26.4) | 25.9 (24.6-29) | 0.795 |
| Hypertension, n (%) | 9 (64.2) | 6 (54.5) | 0.696 |
| Diabetes mellitus, n (%) | 2 (14.2) | 1 (9.0) | 0.691 |
| Active smoking, n (%) | 6 (42.8) | 2 (18.1) | 0.233 |
| GFR (ml/min/1.73m2 ), median (IQR) | 81.5 (72-94) | 72 (44-84) | 0.104 |
| Hemoglobin (g/dl), median (IQR) | 14.2 (13.6-15.4) | 11.1 (10.4-11.5) | 0.0001 |
| STEMI n (%) | 13 (92.8) | 5 (45.4) | 0.133 |
| NSTEMI n (%) | 1 (7.1) | 4 (36.3) | 0.133 |
Supplementary Table 4. Patient characteristics of ACS patients with and without anemia. Chi-square test was used to compare between two categorical variables. Depending on the distribution of the data, either the student’s t-test or the Mann–Whitney U test was used to compare variables between the two groups. BMI: body mass index; IQR: interquartile range; GFR: glomerular filtration rate; n, number of patients. The patient samples listed in this table were used for the experiments presented in Figure 6D–F.
